# Palladium prompted on-demand cysteine chemistry for the synthesis of challenging and uniquely modified proteins

**DOI:** 10.1038/s41467-018-05628-0

**Published:** 2018-08-08

**Authors:** Muhammad Jbara, Shay Laps, Michael Morgan, Guy Kamnesky, Guy Mann, Cynthia Wolberger, Ashraf Brik

**Affiliations:** 1 Schulich Faculty of Chemistry, Technion-Israel Institute of Technology, Haifa, 3200008 Israel; 2 Department of Biophysics and Biophysical Chemistry, Johns Hopkins University School of Medicine, 725 North Wolfe Street, Baltimore, MD 21205-2185 USA

## Abstract

Organic chemistry allows for the modification and chemical preparation of protein analogues for various studies. The thiolate side chain of the Cys residue has been a key functionality in these ventures. In order to generate complex molecular targets, there is a particular need to incorporate orthogonal protecting groups of the thiolated amino acids to control the directionality of synthesis and modification site. Here, we demonstrate the tuning of palladium chemoselectivity in aqueous medium for on-demand deprotection of several Cys-protecting groups that are useful in protein synthesis and modification. These tools allow the preparation of highly complex analogues as we demonstrate in the synthesis of the copper storage protein and selectively modified peptides with multiple Cys residues. We also report the synthesis of an activity-based probe comprising ubiquitinated histone H2A and its incorporation into nucleosomes and demonstrate its reactivity with deubiquitinating enzyme to generate a covalent nucleosome–enzyme complex.

## Abstract (web summary)

Cysteine side chains are reactive sites of protein synthesis and modification. Here, the authors show that tuning palladium chemoselectivity allows for selective removal of several cysteine protection groups, and use their method to synthesize challenging protein analogues.

## Introduction

Organic chemistry has revolutionized the field of protein science by contributing efficient synthetic tools to prepare various protein analogues, such as proteins labeled with fluorophores, D-amino acids and posttranslational modifications (PTMs)^1–3^. These analogues have been utilized in various biochemical, structural, and functional studies. Much of what has been done in the area of modifications of expressed proteins is based on the chemistry of the thiolate side chain of the Cys residue;^1–5^ either by employing direct alkylation/arylation or by transforming it to other functionalities, such as dehydroalanine (DHA)^6^. The latter approach has expanded the chemistry for modifications and the ability to generate protein activity-based probes^7–9^. The chemistry of the Cys side chain has also been critical in both the total chemical synthesis and semisynthesis of proteins^1,10,11^. For example, native chemical ligation (NCL)^12^, which is the most utilized approach for assembling the desired polypeptide sequence, relies on the presence of N-terminal Cys, β-, γ- and δ-thiolated amino acids^1^, or a thiol modified auxiliary^13–16^. The thiol handle in these derivatives promotes trans-thioesterification with a peptide thioester for the subsequent *S-N* acyl transfer and formation of an amide peptide bond at the ligation site.

When considering the multistep synthesis of different protein analogues, there is an intrinsic requirement for incorporating orthogonal protecting groups (PGs) of the thiolated amino acids^17^. The site-specific removal of a particular PG makes it possible to control the direction of synthesis (e.g., side chain branching) and/or the modification site. Several reports have demonstrated the usefulness of NCL coupled with orthogonal protection for the synthesis of highly complex analogues^18–20^. Examples include the synthesis of branched proteins, such as ubiquitinated proteins;^21–23^ proteins containing multiple Cys residues (e.g., analogues of EPO protein);^18,24,25^ and site-specific antibody–drug conjugates^26^. In these cases, orthogonal protection of Cys residues, mainly by using thiazolidine (Thz)^27^ and acetamidomethyl (Acm)^28^, is a critical step in obtaining the product in the desired form^29,30^. Despite the utility of the current toolbox for Cys protection/deprotection, there are still limitations to these strategies. The need for harsh removal conditions, prolonged reaction times and additional HPLC-purifications steps all limit the application of these approaches to more challenging systems. Therefore, expanding the available toolbox to manipulate Cys PGs in an orthogonal manner would enable the synthesis and study of complex protein targets. We recently reported that palladium complexes can remove multiple Cys PGs within minutes in a fully aqueous medium^31–34^, which also could be coupled in situ with NCL conditions to provide excellent yields of the desired products^35,36^. Although these conditions have resulted in important advances in chemical protein synthesis^31–34^, the utility of these approaches is limited by the lack of chemoselectivity of the palladium complexes when applying more than one PG in the synthesis.

Here we demonstrate palladium chemoselectivity in a fully aqueous medium for on-demand deprotection of Thz and Acm protections. We also show efficient removal of Cys *t*-butyl PG using palladium in a fully aqueous medium and demonstrate its stability under the removal conditions of Thz and Acm. The chemistry reported here is used for the one-pot and site-specific modification of peptides containing multiple Cys residues, as well as for the total chemical synthesis of copper storage protein 1 (CSP-1), which contains 13 Cys residues. We also show the total chemical synthesis of an activity-based probe of ubiquitinated histone H2A at Lys119 to prepare ubiquitinated nucleosome core particle probes (NCP-(H2A-Ub_DHA_)). This probe exhibits reactivity with the Calypso/ASX heterodimer deubiquitinase (DUB) to form a stable covalent nucleosome–enzyme complex.

## Results

### Palladium prompted on-demand deprotection of Thz and Acm

**Fig. 1:**
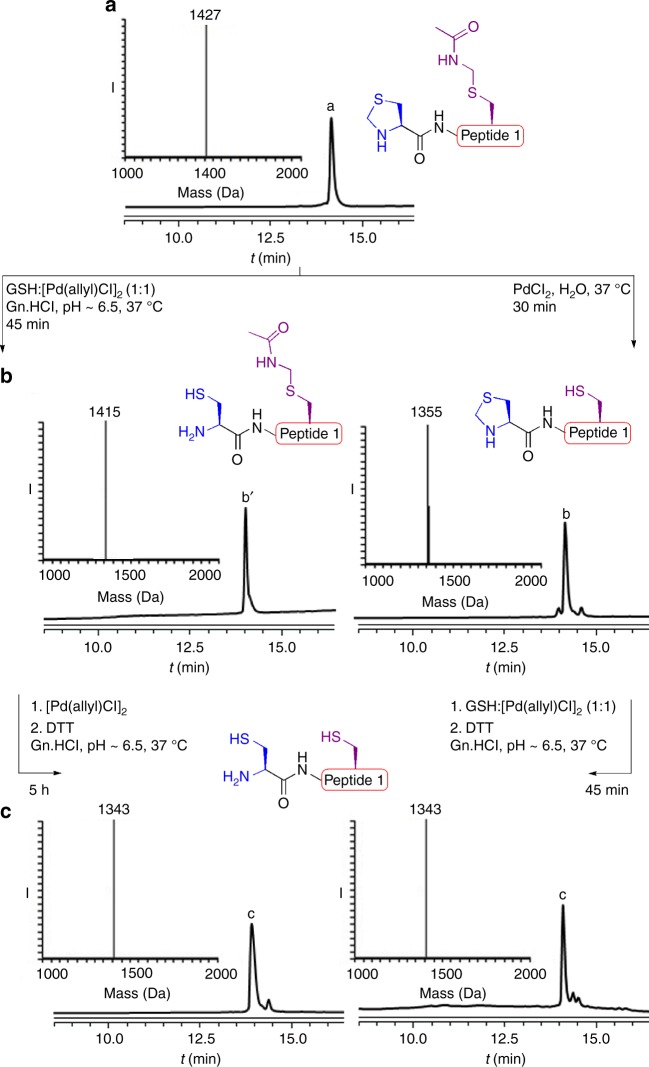
On-demand deprotection of Thz and Acm PGs via palladium complexes. **a** Analytical HPLC and mass traces for protected peptide 1 with the observed mass 1427 ± 0.6 Da, calcd 1427 Da (average isotopes). **b** Analytical HPLC and mass traces for Thz or Acm deprotection via palladium with the observed masses 1415 ± 0.3 Da, and 1355 ± 0.5 Da, calcd 1415 Da and 1356 Da (average isotopes), respectively. **c** Analytical HPLC and mass traces for Acm or Thz deprotection via palladium with the observed masses 1343 ± 0.6 Da, calcd 1344 Da (average isotopes)

We recently found that glutathione (GSH) as an additive can accelerate the cleavage of peptides and proteins connected via a Thz linkage using [Pd(allyl)Cl]_2_ under physiological pH^32^. Notably, when GSH was used in a 1:1 molar ratio to [Pd(allyl)Cl]_2_, we found that breaking the Thz linkage was more effective compared to using 4-mercaptophenylacetic acid (MPAA) and tris(2-carboxyethyl)phosphine (TCEP) additives^31^. This prompted us to revisit the Thz and Acm deprotection via palladium, by synthesizing the model peptide, Thz-LYRAGC(Acm)LYRAG (peptide 1), bearing N-terminus Thz and internal Cys protected with Acm. When this peptide was exposed to [Pd(allyl)Cl]_2_ and GSH (1:1) in 6 M Gn·HCl, pH~6.5 at 37 °C, we observed within 45 min the full unmasking of the Thz to the free N-terminal Cys. Surprisingly, the Acm under these conditions was completely stable. Interestingly, upon addition of an extra 10 equiv. of [Pd(allyl)Cl]_2_ to the reaction mixture, the Acm was completely removed within 5 h (Fig. 1). When examining PdCl_2_, the more efficient palladium complex for the Acm removal with GSH^33^, we observed the complete removal of the Thz and a relatively lower stability of the Acm compared to the reaction with [Pd(allyl)Cl]_2_ and GSH (Supplementary Fig. 2).

The ability to control Cys deprotection with Acm and Thz encouraged us to further investigate the effect of different parameters on the removal of the two PGs. Previous studies have shown the effect of guanidine as a ligand on the palladium reactivity in Suzuki cross-coupling reaction under aqueous conditions^37^. With this in mind, we decided to examine the removal of the two PGs in the absence of guanidine. Here we found that when exposing peptide 1 to PdCl_2_ in water only, we observed reversed and efficient removal of the Acm within 30 min, while the Thz remained completely stable (Fig. 1b). In situ addition of [Pd(allyl)Cl]_2_ and GSH in 6 M Gn·HCl to the reaction mixture enabled subsequent efficient Thz opening within 45 min (Fig. 1c).

To examine the origin of chemoselectivity of the deprotection we kept the two reactions shown in Fig. 1b, after the first removal, for additional time. After 2 h we observed the removal of the Acm or Thz (< 10%). Nevertheless, even after overnight the deprotection of the second protecting group did not reach completion and only 50–60% of the fully deprotected peptide was observed in both cases. This could suggest that these reactions are kinetically controlled. However, one could not exclude the formation of different palladium complexes along the reaction pathway that could eventually affect the reaction chemoselectivity.

### Palladium-mediated Cys(*t*-butyl) deprotection

**Fig. 2:**
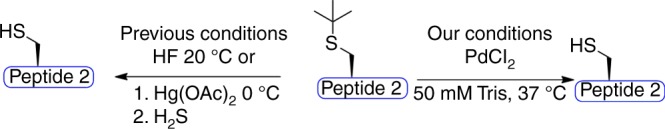
Removal conditions of Cys(*t*-butyl). Schematic representation of *t*-butyl deprotection via PdCl_2_ in aqueous buffer compared to the previous conditions

The impressive on-demand deprotection that was observed between Thz and Acm PGs prompted us to search for additional Cys PGs that could be removed under mild treatment with palladium and in an orthogonal manner to the Thz and Acm. Such a development would further expand the toolbox for complex peptide and protein synthesis and modification. We decided to explore the Cys(*t-*butyl) PG^38^, which has been used for regioselective disulfide bond formation in peptides and proteins. This PG can be removed under extremely harsh conditions, such as trifluoroacetic acid and 2,2-dithiobis(5-nitropyridine), hydrofluoric acid or by using mercury metal in organic solvents^38,39^. We wondered if palladium could assist the removal of the *t-*butyl in an aqueous buffer. In order to test this, we synthesized the model peptide AC(*t*-butyl)LYRAGLYRAG (peptide 2) and exposed it to various removal conditions employing different palladium complexes, additives, and temperatures (Table 1, Fig. 2). We found that the *t*-butyl PG was highly stable in all these conditions, as long as the reaction was performed in a Gn·HCl buffer. However, when screening different buffers, efficient *t*-butyl deprotection was observed within 1.5 h using PdCl_2_ in a 50 mM Tris buffer at 37 °C (Table 1). We also found that performing the same reaction in a urea buffer gave similar results (Table 1), which supports the critical role of guanidine in palladium reactivity.

**Table 1:** Examining *t*-butyl deprotection under various conditions

Entry	Catalyst	Equiv.	Buffer	Additive	Temperature	Time	Yield
1.	[Pd(allyl)Cl]_2_	20	6 M Gn·HCl	GSH	37 °C	5 h	0%
2.	[Pd(allyl)Cl]_2_	10	6 M Gn·HCl	MPAA	37 °C	5 h	0%
3.	[Pd(allyl)Cl]_2_	10	6 M Gn·HCl	TCEP	37 °C	5 h	0%
4.	[Pd(allyl)Cl]_2_	10	6 M Gn·HCl	MPAA/TCEP	37 °C	5 h	0%
5.	[Pd(allyl)Cl]_2_	10	6 M Gn·HCl	–	65 °C	5 h	0%
6.	PdCl_2_	20	6 M Gn·HCl	GSH	37 °C	5 h	0%
7.	PdCl_2_	10	6 M Gn·HCl	MPAA/TCEP	37 °C	5 h	0%
8.	PdCl_2_	10	6 M Gn·HCl	TIS	37 °C	5 h	0%
9.	PdCl_2_	10	6 M Gn·HCl	–	37 °C	5 h	0%
10.	PdCl_2_	10	6 M Gn·HCl	–	65 °C	5 h	0%
11.	PdCl_2_	10	0.5 M Gn·HCl	–	37 °C	5 h	0%
12.	PdCl_2_	10	5 M Urea	–	37 °C	2 h	100%
13.	PdCl_2_	10	50 mM Tris	–	37 °C	1.5 h	100%
14.	–	–	5 M Urea	–	37 °C	2 h	0%
15.	–	–	50 mM Tris	–	37 °C	2 h	0%

*TIS* triisopropylsilane, *Tris* 2-Amino-2-(hydroxymethyl)propane-1,3-diol

To examine the stability of the three PGs with different palladium complexes on a multi-Cys protected peptide, we synthesized the model peptide Thz-LYRAC(Acm)LYRAC(*t*-butyl)LYRAG. By exposing this peptide to [Pd(allyl)Cl]_2_ and GSH in 6 M Gn·HCl, followed by the addition of an extra equivalent of [Pd(allyl)Cl]_2_, we were pleased to observe the sequential removal of Thz and Acm while keeping the *t*-butyl intact (Supplementary Fig. 7).

### Palladium-enabled peptide modifications at selected sites

**Fig. 3:**
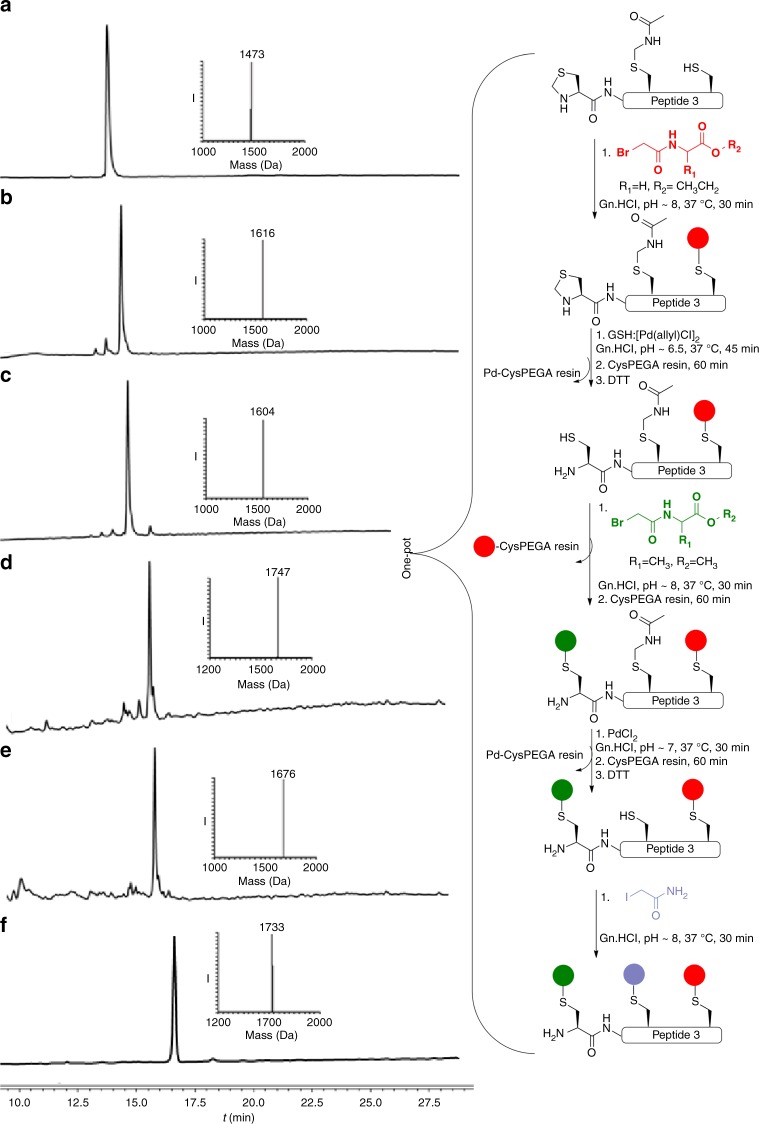
Schematic representation of palladium-mediated modification of peptide 3 at selected Cys sites. **a** Reaction at time zero: main peak corresponds to peptide 3 with the observed mass 1473.0 Da ± 0.4 Da, calcd 1473.7 Da (average isotopes). **b** 1st alkylation reaction, main peak corresponds to mono-alkylated peptide 3 with the observed mass 1616.0 Da ± 0.5 Da, calcd 1616.7 Da (average isotopes). **c** Thz opening: main peak corresponds to Thz-deprotected peptide 3 with the observed mass 1604.0 Da ± 0.7 Da, calcd 1604.7 Da (average isotopes). **d** 2nd alkylation reaction: main peak corresponds to di-alkylated peptide 3 with the observed mass 1747.0 Da ± 0.4 Da, calcd 1747.7 Da (average isotopes). **e** Acm deprotection: main peak corresponds to Acm-deprotected peptide 3 with the observed mass 1676.0 Da ± 0.4 Da, calcd 1676.7 Da (average isotopes). **f** 3rd alkylation reaction: main peak corresponds to tri-alkylated peptide 3 with the observed mass 1733.0 Da ± 0.4 Da, calcd 1733.6 Da (average isotopes). Red sphere: acetylglycine-ethyl ester, green sphere: acetylalanine-methyl ester, purple sphere: acetamide

Encouraged by these results, we turned our efforts to selectively modify a Cys residue within a peptide containing multiple Cys residues by employing selective removal of Cys PGs coupled with alkylation. In principle, this could enable the rapid synthesis of peptide libraries from a common scaffold, which might find useful applications in various areas such as in medicinal chemistry^26^. To explore this concept, we synthesized a model peptide bearing three different Cys residues, Thz-LYRAGC(Acm)LYRCA (peptide 3) for sequential alkylation. To alkylate the free Cys first, we treated peptide 3 with halogenated-acetyl derivative, which was completed within 30 min (Fig. 3b). For the second alkylation, we performed selective Thz deprotection followed by in situ coupling with a second halogenated-acetyl derivative. In this case, however, no alkylation was observed, most likely because the Cys remained bound to palladium in a Pd–S bond, renders it unreactive for the alkylation step. While quenching the reaction with dithiothreitol (DTT) is useful for both the precipitation of excess palladium and for breaking the Pd-S bond^31^ such a step can be problematic for the alkylation reactions, as DTT can react with the halogenated-acetyl derivative. To overcome this obstacle, we utilized a Cys-PEGA resin, which is a water-compatible solid support, to capture any excess of palladium from the reaction mixture, where we can easily separate the reaction mixture from the remaining palladium and without interfering with the halogenated-acetyl derivative applied in the next step (Supplementary Fig. 9). Hence, after Thz deprotection we treated the reaction mixture with the Cys-PEGA resin to capture all the free palladium (Fig. 3c). The reaction mixture was then separated from the resin and treated with one equiv. of DTT, which we found to be more powerful in releasing the bound palladium to the sulfur and enable a second alkylation step (Fig. 3d). In addition, the Cys-PEGA resin could quench any residual halogenated-acetyl derivative in the reaction mixture. Following this step the Acm was removed and the quenching step with the resin was repeated to enable a third alkylation reaction with the iodoacetamide (Fig. 3f). Using this approach, we were able to selectively modify the three Cys residues in peptide 3 in a one-pot operation within 7 h for the 11 steps. The above described sequential deprotection and Cys alkylation on peptide 3 was also attempted in reversed order for the deprotection of the two groups, i.e., first Acm followed by Thz removal for sequential modification. Such an approach was also successful and provided tri-alkylated peptide 3 (Supplementary Fig. 10).

### Palladium-mediated total chemical synthesis of CSP-1

**Fig. 4:**
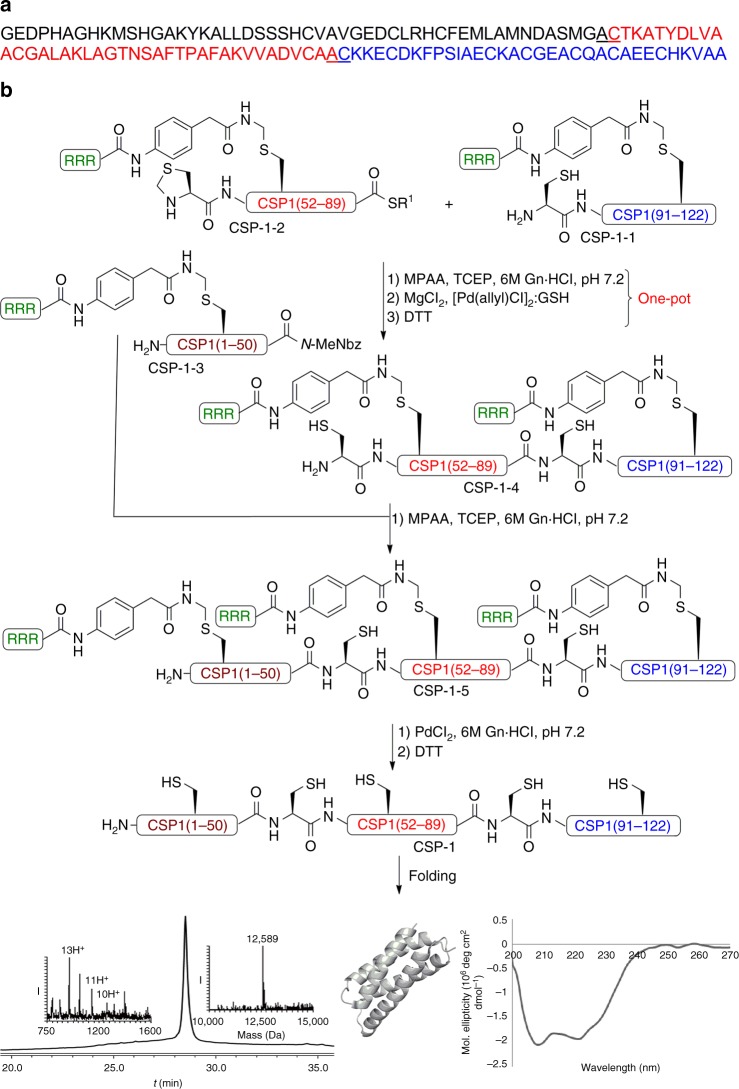
Total chemical synthesis of CSP-1. **a** The CSP-1 sequence where the two-ligation sites are underlined. **b** Synthesis of CSP-1, analytical HPLC and mass traces for purified CSP-1 with the observed mass 12589 ± 1.1 Da, calcd 12590 Da (average isotopes) and CD spectrum. R^1^: 3-Mercaptopropionate, *N*-MeNbz: *N*-acyl-*N*′-methylacylurea

With the results described above, we then tested the applicability of palladium-mediated selective chemistry on Cys residues for the synthesis of complex proteins. We chose the Cys-rich protein CSP-1 as a model system, which contains 13 Cys residues. CSP-1 was very recently discovered as a copper storage protein for particulate methane monooxygenase from the methanotroph, *Methylosinus trichosporium* OB3b^40^. Our efforts to prepare this protein by the synthesis of three peptide fragments coupled with NCL were unsuccessful despite several attempts (Supplementary Fig. 11-13). This is probably due to the protein hydrophobic sequence and the presence of many Cys residues, which renders the peptide fragments hardly soluble during the ligation reactions. To overcome this obstacle, we used the same three fragments, CSP-1-1, CSP-1-2, CSP-1-3 but each was modified temporarily with a phenylacetamidomethyl (Phacm) as a cleavable solubilizing tag^34,41^, bearing three Arg residues to enhance the solubility of the fragments (Fig. 4). The tags were incorporated into the different peptide fragments at positions Cys26, 62, and 117. The three-Arg tags dramatically improved the peptides’ solubility and enabled their preparation in good yields as compared to the standard approach (Supplementary Fig. 14-16). Indeed, the presence of the tags enabled efficient NCL of CSP-1-1 and CSP-1-2. For the second ligation with CSP-1-3, selective Thz opening was necessary, keeping in mind that the solubilizing tags should remain intact until the end of synthesis despite the use of palladium to deprotect the Thz. Since the NCL step requires MPAA and TCEP additives the reactivity of the palladium will be affected and interfere with the desired selectivity, i.e., deprotecting the Thz without cleaving the Phacm linker. To neutralize the effect of MPAA and TCEP on palladium reactivity, we envisioned the use of MgCl_2_ to chelate these additives^33^. Indeed, when we performed NCL between the CSP-1-1 and CSP-1-2 peptides in the presence of MPAA and TCEP, followed by MgCl_2_ treatment and [Pd(allyl)Cl]_2_/GSH, this enabled selective Thz removal without affecting the three Phacm tags to provide CSP-1–4. Subsequently, the second ligation with fragment CSP-1-3 was performed to give CSP-1-5. Finally, the three tags were removed using PdCl_2_ for 1 h to provide the native CSP-1 protein, which showed by circular dichroism (CD) the expected secondary structure (Fig. 4).

### Palladium-mediated chemical synthesis of H2AK119_DHA_Ub

Ubiquitin activity-based probes have found great utility in understanding the function and structure of deubiquitinases^42,43^. We aimed to use the on-demand palladium chemistry to prepare probes based on ubiquitinated proteins. We focused on probes that are based on ubiquitinated H2A at Lys119 (H2AK119Ub) where the ubiquitin carboxyl terminus is covalently linked to the ε-amino group of lysine via an isopeptide bond. In this probe, Gly76 is replaced with DHA to trap the DUB catalytic Cys, as we previously reported for diubiquitin activity-based probes^9^. Ubiquitination of H2A is a hallmark of heterochromatin, which is dynamically regulated by Calypso/ASX in *Drosophila*^44^, and plays an important role in cell cycle control, DNA damage response, and gene regulation^45^. We anticipate that this type of chemistry could provide useful reagents that would assist in understanding the structural and functional mechanisms of Calypso/ASX DUB in chromatin context.

**Fig. 5:**
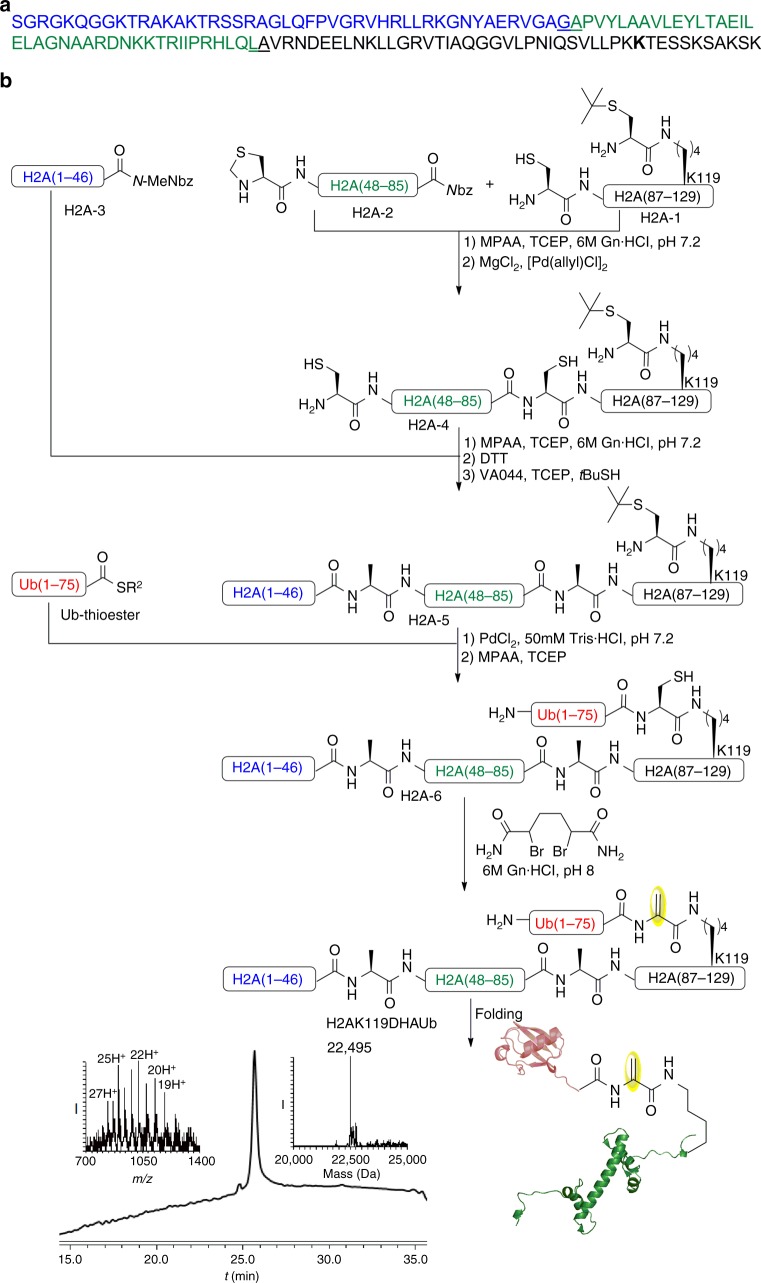
Total chemical synthesis of H2AK119_DHA_Ub. **a** The H2A sequence where the two-ligation sites are underlined. **b** Synthesis of H2AK119_DHA_Ub and analytical HPLC and mass traces for purified H2AK119_DHA_Ub with the observed mass 22495 ± 2.7 Da, calcd 22498 Da (average isotopes), H2A in green, ubiquitin in red. *N*-MeNbz: *N*-acyl-*N*-methylacylurea, *N*bz *N*-acyl-benzimidazolinone, R^2^ 3-Mercaptopropionate, VA044 2,2’-Azobis[2-(2-imidazolin-2-yl)propane] Dihydrochloride

For the total chemical synthesis of the H2AK119Ub probe, we prepared three peptide fragments based on the H2A sequence (H2A-1, H2A-2, and H2A-3) in addition to Ub(1-75)-thioester (Fig. 5)^36,46^. To enable ubiquitination through Lys119 and latent DHA formation, fragment H2A-1 was prepared bearing also Cys(*t*-butyl) on Lys119. This Cys, which replaces Gly76 of Ub would serve after H2A polypeptide assembly for the ligation with Ub and DHA formation. Here we envisioned selective removal of the Thz for sequential backbone ligation in the presence of the orthogonally protected Cys. In this synthesis, Ala at positions 86 and 48 in H2A-2 and H2A-3 fragments were temporarily mutated to Cys to enable NCL. With this design in mind, we performed the first ligation between H2A-1 and H2A-2, followed by MgCl_2_ treatment to enable Thz removal via [Pd(allyl)Cl]_2_. Subsequently, this enabled a second NCL step with fragment H2A-3 to provide H2A(1-129)K119-C(*t*-butyl). At this stage, we performed a desulfurization step in a one-pot operation without affecting the *t*-butyl protection to give the full-length H2A-5. The purified intermediate H2A-5 was dissolved in urea or Tris buffer at pH 7.5 and treated with PdCl_2_ to unmask the Cys(*t-*butyl) to give H2A(1–129)K119Ub-Cys and was ligated with Ub(1–75)-thioester to furnish the ubiquitinated H2A-6. Subsequently, treatment with the bisamide1,4-dibromobutane^47^ converted the Cys side chain at the ligation site to DHA to provide the desired H2AK119_DHA_Ub probe (Fig. 5).

### Calypso/ASX DUB labeling with NCP-(H2A-Ub_DHA_)

Having the synthetic probe in hand, we sought to demonstrate that the reactive probe could crosslink with the deubiquitinating enzyme complex known to cleave ubiquitin from H2A, Calypso/ASX^44^. We reconstituted the H2A-Ub probe, H2B, H3, H4 and the 146 base pair Widom 601 DNA sequence^48^ into nucleosome core particles (NCP) using standard methods^49^. Under these conditions, we observed negligible labeling with Calypso/ASX DUB, perhaps suggesting that chemical modification of the DHA moiety may be producing an inactive species. To test this, we then moved to examine the probe stability in the presence of several solution components present during NCP reconstitution, including different buffers (Tris and HEPES), additives (TCEP), and temperatures (4 °C, room temperature and 37 °C). To study this, we synthesized a ubiquitinated C-terminal H2A peptide probe (H2A(116-129)K119_DHA_Ub) (Supplementary Fig. 25). HPLC and mass analysis showed that under standard conditions, TCEP reacts with the DHA residue such that > 50% of the probe is deactivated within 24 h, probably via the formation of a stable C-P bond (Supplementary Fig. 27-30)^50,51^. We reasoned that excluding TCEP from the buffer would provide greater stability of the probe for several weeks.

**Fig. 6:**
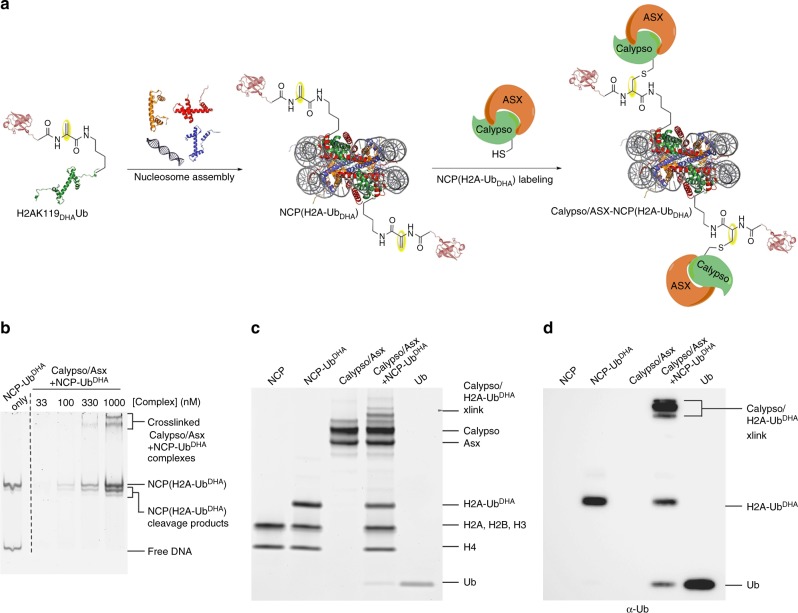
NCP(H2A-Ub_DHA_) labeling with Calypso/ASX DUB. **a** Schematic of nucleosome probe generation and labeling with Calypso/ASX DUB. H2A in green, ubiquitin in light red, H4 in orange, H3 in blue, H2B in red and DNA in gray **b** EMSA analysis suggests the formation of a stable product, even in serial dilutions **c** SDS-PAGE analysis of the labeling reaction between NCP(H2A-Ub_DHA_) and Calypso/ASX DUB reveals the generation of a band with higher molecular weight, corresponding to a Calypso/ASX-H2A-Ub_DHA_ crosslinked species. **d** Western blot analysis of samples shown in (c) using ubiquitin antibody shows high-molecular-weight species, which contains ubiquitin formed upon reaction of Calypso/ASX with NCP(H2A-Ub_DHA_)

With the insight that TCEP inactivates DHA reactivity, we reconstituted the H2A-Ub_DHA_ probe together with recombinant *Xenopus laevis* histones H3, H4, H2B and the Widom 601 DNA sequence as described above, but in the absence of reducing agent. Histones were reconstituted into octamers and purified by size-exclusion chromatography to remove incorrectly folded histone complexes^52^. The histone octamers were further reconstituted into NCPs by gradient salt dialysis in the presence of the Widom 601 DNA purified by standard methods to provide NCP(H2A-Ub_DHA_) probe (Fig. 6a). The NCP(H2A-Ub_DHA_) probe was incubated for 1 h at 25 °C with Calypso/ASX at concentrations of 3 µM and 6 µM, respectively. The resultant products were analyzed by electrophoretic mobility shift assay (EMSA), SDS-PAGE, and Western blot. Taken together, these results indicate the formation of a unique covalent complex was formed between Calypso/ASX and NCP-Ub_DHA_. The EMSA experiment showed the formation of a band corresponding to nucleosomal DNA migrating at a higher apparent molecular weight than NCP-Ub_DHA_ alone. We also observed no dissociation of this complex after serial dilution of the reaction mix supporting the formation of enzyme stably bound to the nucleosome probe (Fig. 6b). In addition, an SDS-PAGE denaturing gel similarly showed the formation of a high-molecular-weight species appearing after Calypso/ASX was incubated with NCP(H2A-Ub_DHA_), consistent with the formation of Calypso/ASX-H2A-Ub_DHA_ complex (Fig. 6c). To further validate this band in Fig. 6c was, in fact, a reaction product resulting from labeling by the probe, we performed immunoblotting with an antibody against ubiquitin, which confirmed that the ubiquitin is migrating with an high molecular weight due to covalent cross-linking of NCP(H2A-Ub_DHA_) to the Calypso/ASX DUB (Fig. 6d).

## Discussion

In conclusion, we have introduced synthetic approaches to expand the chemical toolbox for synthesizing and modifying peptides and proteins. The reported approaches make possible on-demand palladium-mediated decaging of various Cys PGs to facilitate peptide modification and protein synthesis of complex targets. Remarkably, the selectivity has been achieved by simple variation of the additives and the solvent in the reaction mixture containing commercially available palladium complexes. The palladium tunability that has been achieved here should also initiate other research directions in aqueous-based chemistry of small molecules and trigger mechanistic studies. The synthesis of protein targets, such as the CSP-1 and the activity-based probes based on ubiquitinated H2A, pave the way for answering fundamental questions related to these systems. Of particular interest is the Calypso/ASX-NCP(H2A-Ub_DHA_) complex, which has opened up the opportunity for structural studies that would otherwise be very difficult to attempt.

## Methods

### Palladium prompted Thz removal in presence of Acm

0.5 mg of peptide 1 (0.35 × 10^−3^ mmol, 2 mM) was dissolved at 175 µL, 6 M Gn·HCl, 200 mM Na_2_HPO_4_ buffer pH 6.5, and treated with 10 equiv. of [Pd(allyl)Cl]_2_ and GSH (1:1) and incubated at 37 °C for 45 min to observe complete Thz opening. Subsequent addition of extra 10 equiv. of [Pd(allyl)Cl]_2_ to the reaction mixture led to complete removal of the Acm within 5 h.

### Palladium prompted Acm removal in presence of Thz

0.5 mg of peptide 1 (0.35 × 10^−3^ mmol, 2 mM) was dissolved at 175 µL, H_2_O, and treated with 10 equiv. of PdCl_2_ and incubated at 37 °C for 30 min to observe complete Acm deprotection. Subsequent addition of [Pd(allyl)Cl]_2_ and GSH (1:1) in 6 M Gn·HCl, 200 mM Na_2_HPO_4_ buffer pH 6.5 to the reaction mixture at 37 °C led to complete Thz removal within 45 min.

### Sequential modification of peptide 3

2 mg of peptide 3 (1.35 × 10^−3^ mmol, 2.7 mM) was dissolved at 500 µL, 6 M Gn·HCl, 200 mM Na_2_HPO_4_ buffer pH 8, and treated with 1 equiv. of *N*-(bromoacetyl)glycine-ethyl ester, which pre-dissolved as a stock solution in MeOH. 100 equiv. of NaI was added to accelerate the alkylation reaction. The reaction was monitored by HPLC-MS, which showed completion within 30 min. Then, the reaction mixture was treated with 10 equiv. of [Pd(allyl)Cl]_2_ and GSH (1:1) and incubated at 37 ° for 45 min to observe complete Thz removal. For the second alkylation, the solution mixture was treated with Cys-PEGA resin for 1 h at 37 °, followed by the addition of 1 equiv. of DTT. Subsequently, 11 equiv. of the *N*-(bromoacetyl)alanine-methyl ester dissolved in MeOH and 100 equiv. NaI were added and incubated at 37 °. The reaction was completed within 30 min. For Acm removal, 10 equiv. of PdCl_2_ was added to the reaction mixture and incubated for 30 min at 37 ° for a complete Acm removal. Then the solution mixture was treated with 40 equiv. Cys-PEGA resin for 1 h at 37 ° and 1 equiv. of DTT. Finally, 2 equiv. of iodoacetamide at 37 ° were added to give the tri-alkylated peptide 3.

### Total chemical synthesis of CSP-1

The peptides CSP-1-1 (3.5 mg, 8.5 × 10^−4^ mmol) and CSP-1-2 (4.3 mg, 9.5 × 10^−4^ mmol) were dissolved in 6 M Gn·HCl, 200 mM Na_2_HPO_4_ buffer, pH ~7.2 (400 µL, 2 mM) containing 20 equiv. of MPAA and 10 equiv. of TCEP. The reaction was incubated at 37 °C for 2 h. Progress of the reaction was monitored by analytical HPLC using C4 analytical column and a gradient of 0–60% buffer B (Acetonitrile mixed with 0.1% TFA) over 30 min. After completion of the ligation reaction, the product was treated with 100 eq. of MgCl_2_ dissolved in 100 µL of 6 M Gn·HCl, 200 mM Na_2_HPO_4_ buffer, pH ~7.2 at 37 °C for 10 min. For Thz removal, [Pd(AllylCl)]_2_ (4.1 mg, 1.3 × 10^−2^ mmol) and GSH (3.8 mg, 1.3 × 10^−2^ mmol) were dissolved in 100 μl of 6 M Gn·HCl, 200 mM Na_2_HPO_4_ buffer, pH ~7.2, and added to the reaction mixture, which was incubated at 37 °C for 1.5 h. Progress of the reaction was monitored by analytical HPLC using C4 analytical column and a gradient of 0–60% B over 30 min. After completion of the reaction, 60 equiv. of DTT was added to quench and precipitate the palladium from the reaction mixture. After centrifugation, the supernatant solution was collected and purified using semi-preparative HPLC, C4 column and gradient of 20–60% B to give the desired product in ~45% yield (Supplementary Fig. 17). For the second ligation, the peptides CSP-1–4, (3.2 mg, 3.8 × 10^−4^ mmol) and CSP-1-3 (2.5 mg, 4.2 × 10^−4^ mmol) were dissolved in 100 μl of 6 M Gn·HCl, 200 mM Na_2_HPO_4_ buffer, pH ~7.2, containing 20 equiv. MPAA and 10 equiv. of TCEP at pH ~7.2 and incubated at 37 °C for 6 h. Progress of the reaction was monitored by analytical HPLC using C4 analytical column and a gradient of 0–60% B over 30 min. After completion of the ligation reaction, CSP1-5 was isolated using semi-preparative HPLC, C4 column and a gradient of 20–60% B to give the desired product in ~50% yield, (Supplementary Fig. 18A).

### Palladium-mediated tag removal

Stock solution of 10 eq. PdCl_2_ was prepared in 6 M Gn·HCl, 200 mM Na_2_HPO_4_ buffer, pH~7.2. Subsequently, the palladium solution was added to the pre-dissolved CSP-1–5, (2.5 mg, 1.7 × 10^−4^ mmol) in 6 M Gn·HCl, 200 mM Na_2_HPO_4_ buffer, pH~7.2 (180 µL, 1 mM) and incubated at 37 °C for 2 h. Progress of the reaction was monitored by analytical HPLC using C4 analytical column with a gradient of 0–60% B over 60 min. After completion of the reaction, 40 equiv. of DTT was added to quench and precipitate the free palladium from the reaction mixture. After centrifugation of the reaction, the supernatant solution was collected purified using semi-preparative HPLC, C4 column and gradient of 20–60% B to give the desired product in ~50% yield, (Supplementary Fig. 18B).

### H2A-5 synthesis

H2A-1 (5 mg, 1.0 × 10^−3^ mmol) and H2A-2, (4.7 mg, 1.0 × 10^−3^ mmol), were dissolved in 6 M Gn·HCl, 200 mM Na_2_HPO_4_ buffer, pH~7.2 (500 µL, 2 mM) containing 20 equiv. of MPAA and 10 equiv. of TCEP. The reaction was incubated at 37 °C for 4 h. Progress of the reaction was monitored by analytical HPLC using C4 analytical column and a gradient of 0–60% B over 30 min. After completion of the ligation reaction, the product was treated with 100 equiv. of MgCl_2_, dissolved in 100 µL of 6 M Gn·HCl, 200 mM Na_2_HPO_4_, pH ~7.2 at 37 °C for 10 min. For Thz removal [Pd(AllylCl)]_2_ (4.9 mg, 1.5 × 10^−2^ mmol) was dissolved in 100 μl of 6 M Gn·HCl, 200 mM Na_2_HPO_4_, pH~7.2. and the reaction mixture was incubated at 37 °C for 1.5 h. Progress of the reaction was monitored by analytical HPLC using C4 analytical column and a gradient of 0–60% B over 30 min. After completion of the reaction H2A-3 fragment (5.7 mg 1.1 × 10^−3^ mmol), dissolved in 100 μl of 6 M Gn·HCl, 200 mM Na_2_HPO_4_, pH~7.2 containing 100 equiv. of MPAA and 50 equiv of TCEP was added to the reaction and incubated at 37 °C for 8 h. Progress of the reaction was monitored by analytical HPLC using C4 analytical column with a gradient of 0–60% B over 30 min. After completion of the final ligation, 60 equiv. of DTT was added to quench and precipitate the palladium from the reaction mixture. After centrifugation of the reaction, the supernatant solution was collected and dialyzed 500 ml of 6 M Gn·HCl, 200 mM Na_2_HPO_4_ buffer at pH~7.2, using Slide-A-Lyzer dialysis cassettes for 16 h. After the dialysis, radical desulfurization reaction was performed by adding 70 mg of TCEP (0.25 M), 32 mg of VA-044 (50 equiv. per sulfur) and 100 μl of *t*-butyl thiol (10% of the total volume) to the reaction mixture and incubated in 42 °C for 4 h. Progress of the reaction was monitored by analytical HPLC using C4 analytical column with a gradient of 0–60% B over 30 min. After centrifugation, the supernatant solution was collected and isolated via semi-preparative HPLC, C4 column and a gradient of 20–60%B was used to isolate the product in ~17% yield, (Supplementary Fig. 22).

### *t*-Butyl removal followed by chemical ubiquitination of H2A

PdCl_2_ (3 mg, 1.7 × 10^−3^ mmol) was dissolved in 100 µL H_2_O and 10 µL of this solution was added to the pre-dissolved H2A-5 (2.5 mg, 1.7 × 10^−4^ mmol) in 50 mM Tris buffer, pH 7.5 (190 µL, 2 mM) and incubated at 37 °C for 2 h. Progress of the reaction was monitored by analytical HPLC using C4 analytical column and a gradient of 0–60% B over 30 min. After completion of *t*-butyl removal, Ub(1–75)-thioester, (2.2 mg, 2.55 × 10^−3^ mmol), was dissolved in 100 µL of 6 M Gn·HCl, 200 mM Na_2_HPO_4_ buffer containing 100 equiv. of MPAA and 50 equiv. of TCEP, pH ~7.2. The reaction was incubated at 37 °C for 4 h. Progress of the reaction was monitored by analytical HPLC using C4 analytical column and a gradient of 0-60% B over 30 min. After completion of the final ligation reaction, 40 equiv. of DTT was added to quench and precipitate the palladium from the reaction mixture. After centrifugation, the supernatant solution was collected and isolated via semi-preparative HPLC, C4 column and a gradient of 20–60% B was used to isolate the product in ~45% yield, (Supplementary Fig. 23).

### H2A-6 conversion to (H2AK119_DHA_Ub) probe

100 equiv. of bisamide1,4-dibromobutane dissolved in DMF were added to H2A-6, (1.2 mg, 8.5 × 10^−5^ mmol) dissolved in 6 M Gn·HCl, 200 mM Na_2_HPO_4_ buffer, pH ~8 (85 µL, 1 mM) and the reaction mixture was incubated 1 h at room temperature, followed by 3 h at 37 °C. Progress of the reaction was monitored by analytical HPLC using C4 analytical column and a gradient of 0-60% buffer B over 30 min. After completion of elimination reaction, the product (H2AK119_DHA_Ub) was purified using semi-preparative HPLC, C4 column and a gradient of 20-60% B was used to give the product in ~55% yield, (Supplementary Fig. 24).

### Nucleosome reconstitution

Histone octamers were reconstituted and purified as described previously^52^ from recombinant *Xenopus Laevis* histone sequences (H2AK119_DHA_Ub, H2B, H3, and H4). The octamers bearing the H2AK119_DHA_Ub were then reconstituted into nucleosomes with salt gradient dialysis and purified using a DEAE-5PW HPLC column (TOSOH). After purification, nucleosomes were dialyzed into NCP storage buffer (10 mM Tris-HCl pH 7.5, 50 mM KCl) and stored at 4 °C.

### Nucleosome labeling

Reaction of Calypso/ASX with the NCP-(H2A-Ub_DHA_) probe was accomplished by reacting each component at concentrations of 6 µM and 3 µM, respectively. The reaction was done in the absence of reducing agent to avoid modification of the DHA group at 25 °C for 1 h in NCP storage buffer. Samples were then analyzed by EMSA (6% TBE gel stained with SYBR Gold), SDS-PAGE (NuPage 4–12% Bis-Tris gel stained with Sypro Ruby), and western blot (antiUb-p4d1 primary (Santa Cruz Biotech Cat # B1512) and anti-mouse IgG HRP-linked secondary (Cell Signaling Cat #: 7076) were diluted at 1:5000, (Supplementary Fig. 31).

### Antibodies

AntiUb p4d1 primary (Santa Cruz Biotech Cat # B1512) and anti-mouse IgG HRP-linked secondary (Cell Signaling Cat #: 7076) were diluted at 1:5000.

### Data availability

The authors declare that all data supporting the findings of this study are available within the article and its Supplementary Information Files, and from the corresponding author upon reasonable request.

## Electronic supplementary material

Supplementary Information
