# Supplementary material for: Palladium prompted on-demand cysteine chemistry for the synthesis of challenging and uniquely modified proteins
Source: Nat Commun. 2018 Aug 8;9:3154. doi: 10.1038/s41467-018-05628-0 (PMC6082840; doi:10.1038/s41467-018-05628-0)
Supplement: Supplementary file 1 — Supplementary Information [file 41467_2018_5628_MOESM1_ESM.pdf]

SUPPLEMENTARY INFORMATION

Palladium Prompted On-Demand Cysteine Chemistry for the Synthesis of Challenging and Uniquely Modified Proteins

Jbara et al.

Supplementary Figures

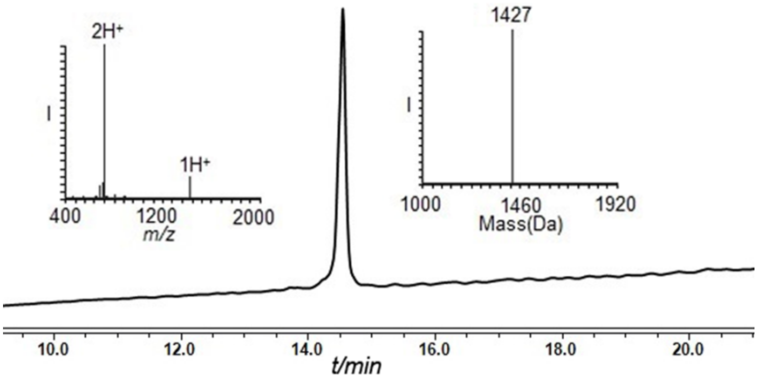

Supplementary Figure 1. HPLC and mass analyses of the purified peptide 1. Observed mass 1427 ± 0.6 Da (calcd 1427.6 Da, average isotopes).

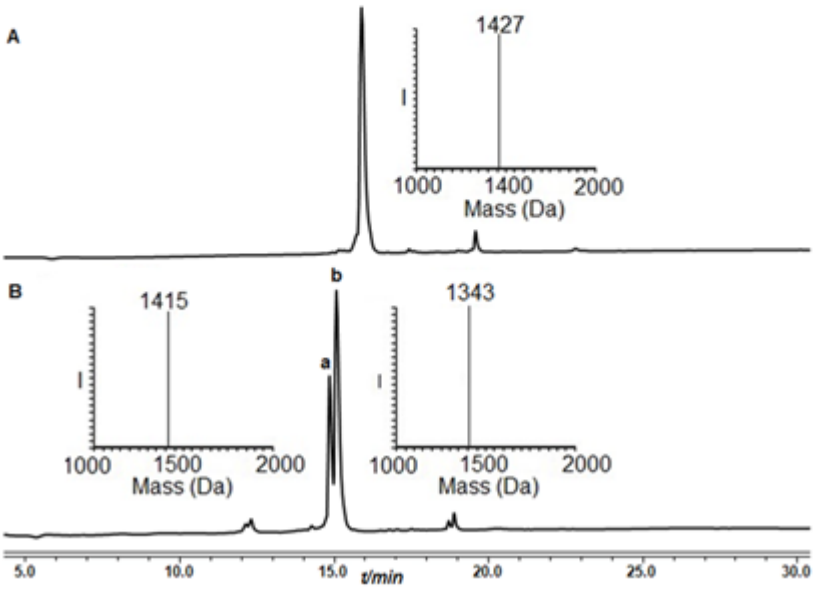

Supplementary Figure 2. HPLC and mass analyses of model peptide 1 using $PdCl_2$:GSH (1:1). (A) at time zero: the peak corresponds to starting material with the observed mass 1427.0 Da ± 0.5 Da (calcd 1427.6 Da, average isotopes). (B) After overnight reaction: peak a corresponds to the Thz opening with the observed mass 1415.0 Da ± 0.3 Da (calcd 1415.6 Da, average isotopes). Peak b corresponds to the Thz and Acm deprotection product with the observed mass 1343.0 Da ± 0.2 Da (calcd 1344.6 Da, average isotopes).

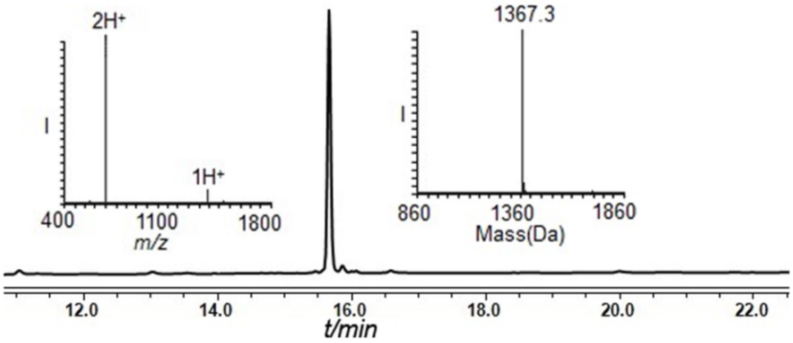

Supplementary Figure 3. HPLC and mass analyses of the purified model peptide 2. Observed mass 1367.3 ± 0.4 Da (calcd 1368.5 Da, average isotopes).

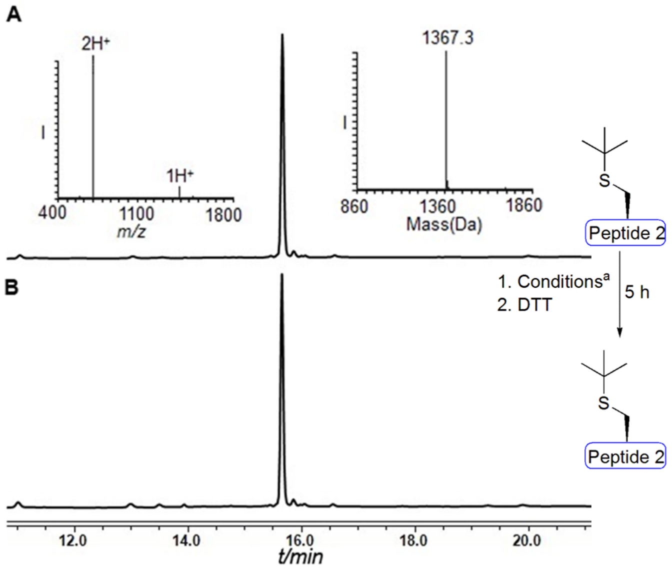

Supplementary Figure 4. HPLC and mass analyses of the stability of Cys(*t*-butyl). (A) Time zero: peak corresponds to model peptide 2, with the observed mass 1367.3 ± 0.4 Da (calcd 1368.5 Da, average isotopes). (B) After treatment with the above mentions conditions: the peak corresponds to the starting material with mass of 1367.3 ± 0.2 Da (calcd 1368.5 Da, average isotopes). [a]Reactions conditions are represented in Supplementary Table 1.

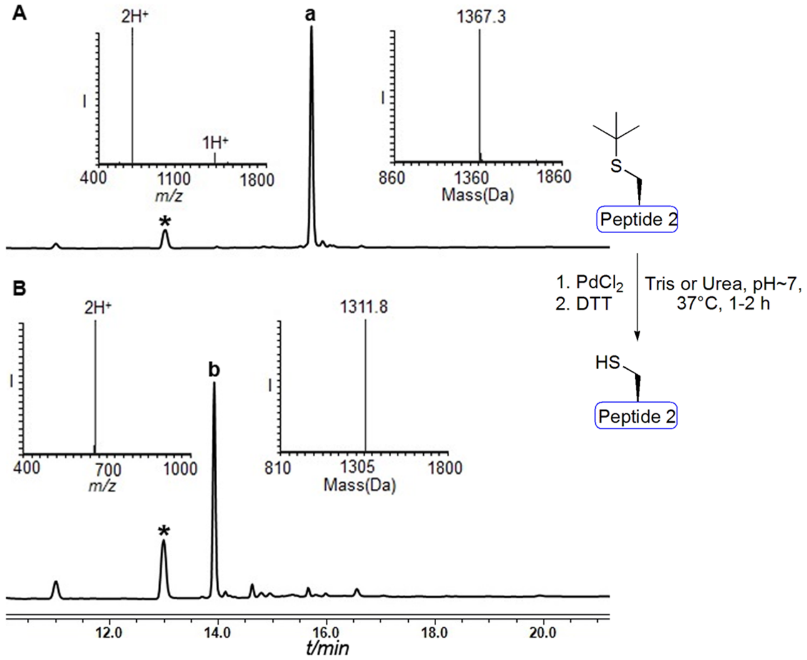

Supplementary Figure 5. HPLC and mass analysis of Cys(*t*-butyl) deprotection via $PdCl_2$ in Tris buffer. (A) Time zero: peak a corresponds to model peptide 2, with the observed mass 1367.3 ± 0.4 Da (calcd 1368.5 Da, average isotopes). (B) After 1.5 h treatment with 10 equiv. $PdCl_2$ in 50mM Tris buffer, 37°C: peak b corresponds to *t*-Butyl removal with the observed mass 1311.8 ± 0.2 Da (calcd 1312.5 Da, average isotopes). *corresponds to deprotected *t*-butyl group to form *t*-butanol with the observed mass 74 ± 0.1 Da (calcd 74 Da, average isotopes).

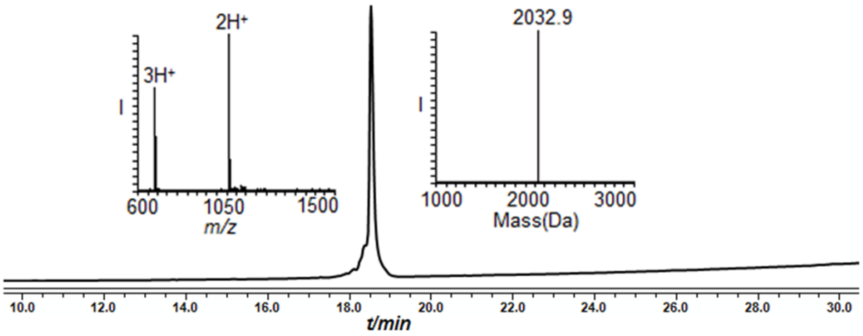

Supplementary Figure 6. HPLC and mass analyses of the purified peptide ThzLYRAC(Acm)LYRAC(*t*-Butyl)LYRAG. Observed mass 2032.9 ± 0.4 Da (calcd 2033.3 Da, average isotopes).

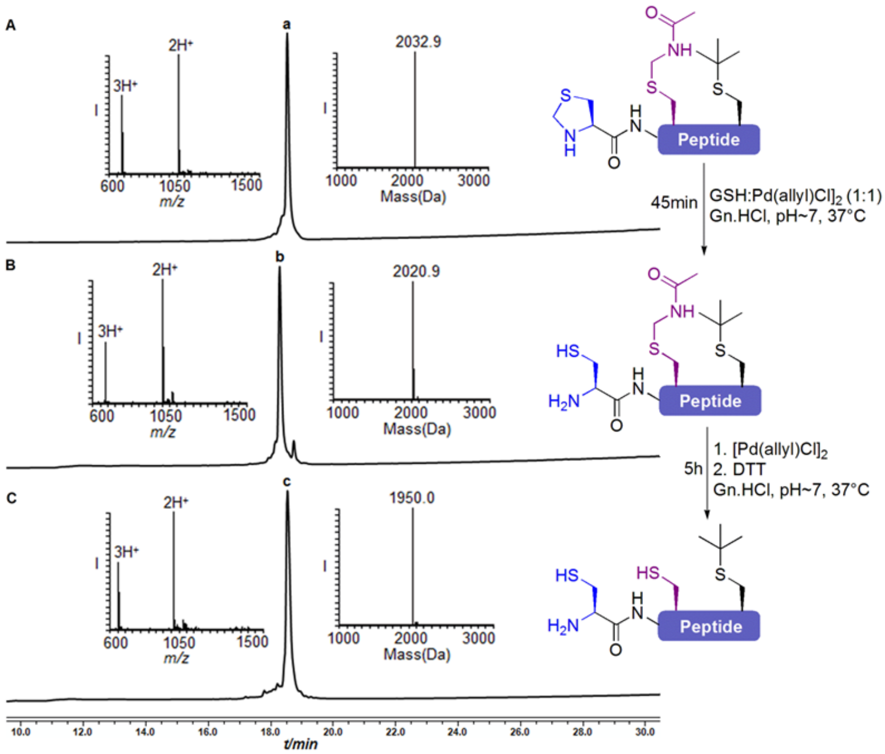

Supplementary Figure 7. HPLC-MS analysis of orthogonal deprotection of peptide containing three protecting groups (Thz, Acm and *t*-Butyl). (A) Time zero: peak a corresponds to the peptide Thz-LYRAC(Acm)LYRAC(*t*-butyl)LYRAG, with the observed mass 2032.9 ± 0.4 Da (calcd 2033.3 Da, average isotopes). (B) After 45 min treatment with 10 equiv. $[Pd(allyl)Cl]_2$ and GSH in 1:1 ratio in 6 M Gn.HCl: peak b corresponds to the Thz removal with the observed mass of 2020.9 ± 0.1 Da (calcd 2021.3 Da, average isotopes). (C) After 5 h treatment with another 10 equiv. $[Pd(allyl)Cl]_2$ in 6 M Gn.HCl, in situ: peak c corresponds to the orthogonal Acm removal with the observed mass 1950.0 ± 0.2 Da (calcd 1950.3 Da, average isotopes).

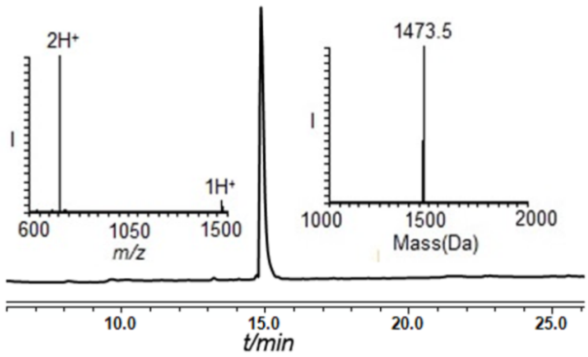

Supplementary Figure 8. HPLC and mass analysis of the purified peptide Thz-LYRAGC(Acm)LYRCA peptide. Observed mass 1473.5 Da ± 0.4 Da (calcd 1473.7 Da, average isotopes).

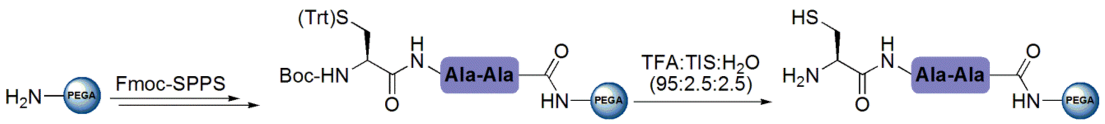

Supplementary Figure 9. Schematic representation of Cys-PEGA resin preparation. The modified resin was prepared by applying standard Fmoc-SPPS strategy.

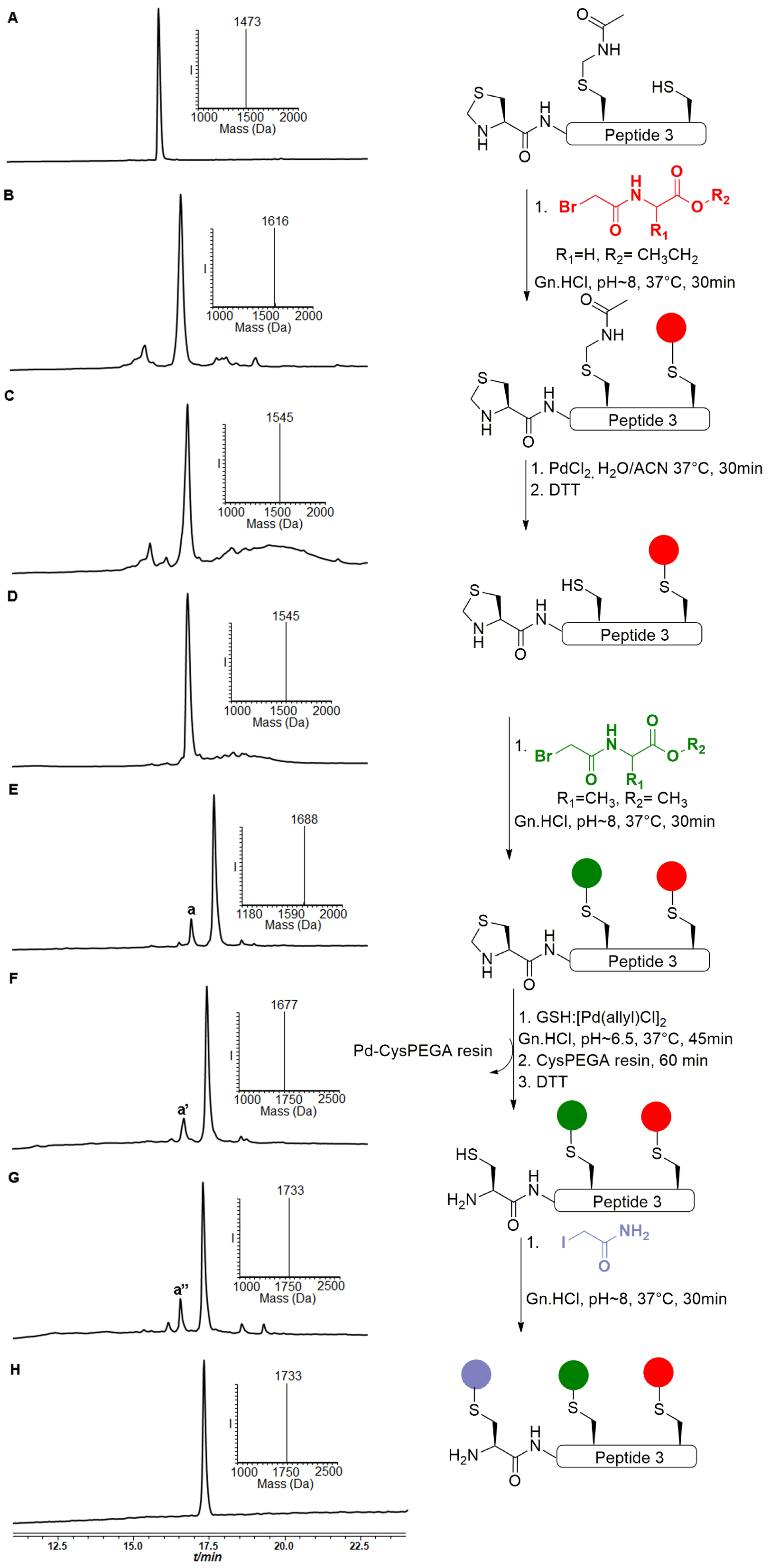

Supplementary Figure 10. Schematic representation of palladium mediated peptide 3 modifications at selected Cys sites. (A) reaction at time zero: the peak corresponds to peptide 3 with the observed mass 1473.0 Da ± 0.1 Da, calcd 1473.7 Da (average isotopes). B) 1st alkylation reaction: the main peak corresponds to mono-alkylated peptide 3 with the observed mass 1616.0 Da ± 0.3 Da, calcd 1616.7 Da (average isotopes). C) Acm deprotection: the main peak corresponds to Acm-

deprotected peptide 3 with the observed mass 1545.0 Da ± 0.3 Da, calcd 1545.7 (average isotopes). D) Purified mono-alkylated peptide 3 after Acm removal with the observed mass 1545.0 Da ± 0.3 Da, calcd 1545.7 (average isotopes). E) 2nd alkylation reaction: the main peak corresponds to di-alkylated peptide 3 with the observed mass 1688.0 Da ± 0.2 Da, calcd 1688.7 Da (average isotopes). Peak a corresponds unalkylated starting material with the observed mass 1545.0 Da ± 0.3 Da, calcd 1545.7 (average isotopes). F) Thz deprotection: main peak corresponds to di-alkylated peptide 3 after Thz opening with the observed mass 1677.0 Da ± 0.2 Da, calcd 1677.7 Da (average isotopes). Peak a' corresponds to deprotected Thz of unalkylated starting material with the observed mass 1533.0 Da ± 0.1 Da, calcd 1533.7 (average isotopes). G) 3rd alkylation reaction: main peak corresponds to tri-alkylated peptide 3 with the observed mass 1733.0 Da ± 0.4 Da, calcd 1733.7 Da (average isotopes). Peak a'' corresponds to alkylated starting material with the observed mass 1647.0 Da ± 0.1 Da, calcd 1647.7 (average isotopes). H) Purified tri-alkylated peptide 3 with the observed mass 1733.0 Da ± 0.4 Da, calcd 1733.7 Da (average isotopes).

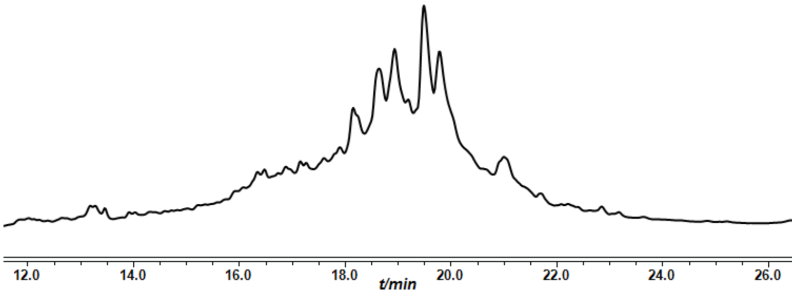

Supplementary Figure 11. HPLC analysis of CSP-1-1 fragment. The peptide was synthesized without the solubilizing tag.

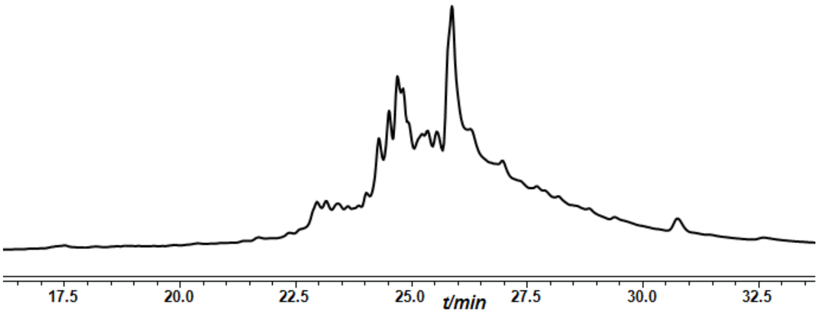

Supplementary Figure 12. HPLC analysis of CSP-1-2 fragment. The peptide was synthesized without the solubilizing tag.

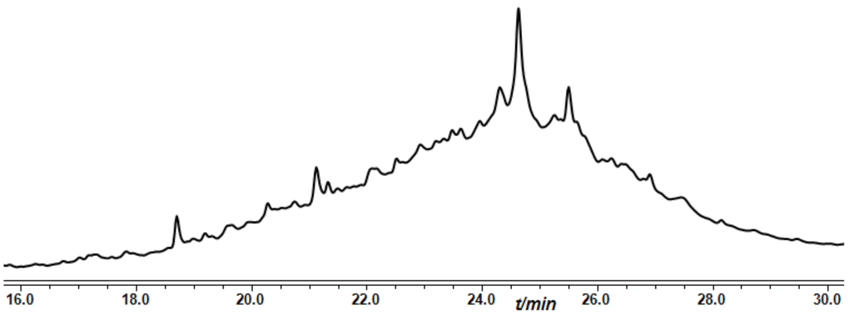

Supplementary Figure 13. HPLC analysis of CSP-1-3 fragment. The peptide was synthesized without the solubilizing tag.

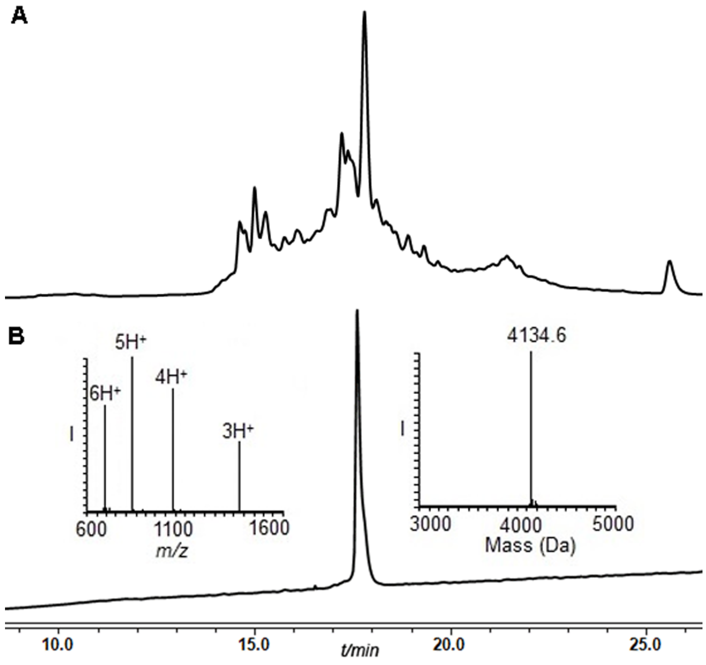

Supplementary Figure 14. HPLC and mass analysis of fragment [Cys(91-122)] with the solubilizing tag (CSP-1-1) (A) crude and (B) purified Csp-1-1 with the observed mass 4134.6 Da ± 0.2 Da (calcd 4135.6 Da, average isotopes).

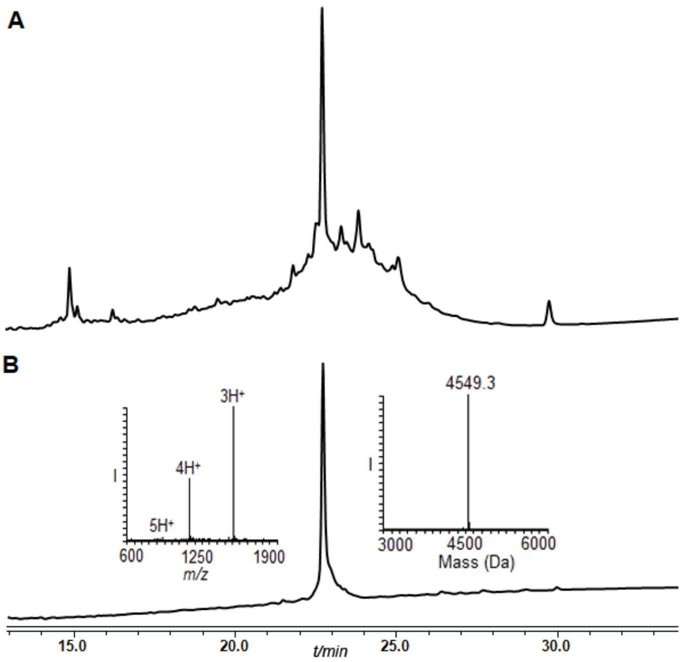

Supplementary Figure 15. HPLC and mass analyses of fragment [Thz-(52-89)-MMP] with the solubilizing tag (CSP-1-2). (A) crude and (B) purified CSP-1-2 with the observed mass 4549.3 Da ± 0.5 Da (calcd 4550.4 Da, average isotopes).

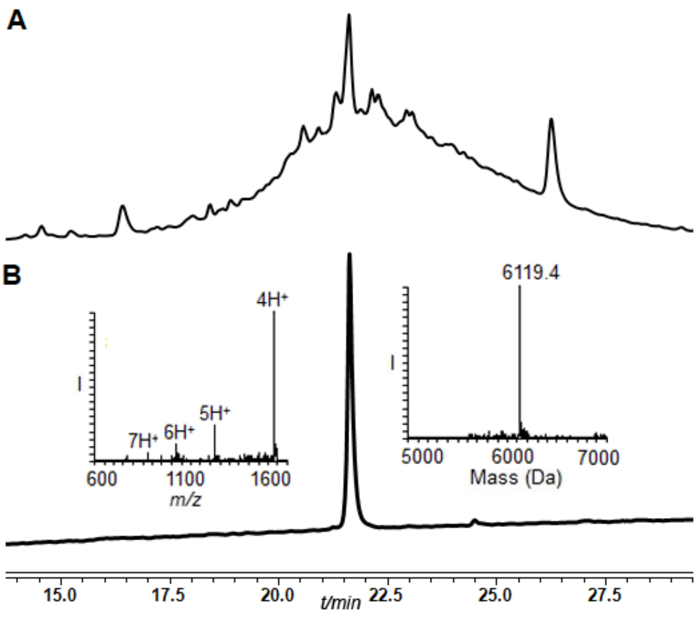

Supplementary Figure 16. **HPLC and mass analysis of fragment [(1-50)-*N*-MeNbz] with the solubilizing tag (CSP1-1-3).** (A) crude and (B) purified CSP-1-3 with the observed mass 6119.4 Da ± 0.3 Da (calcd 6119.5 Da, average isotopes).

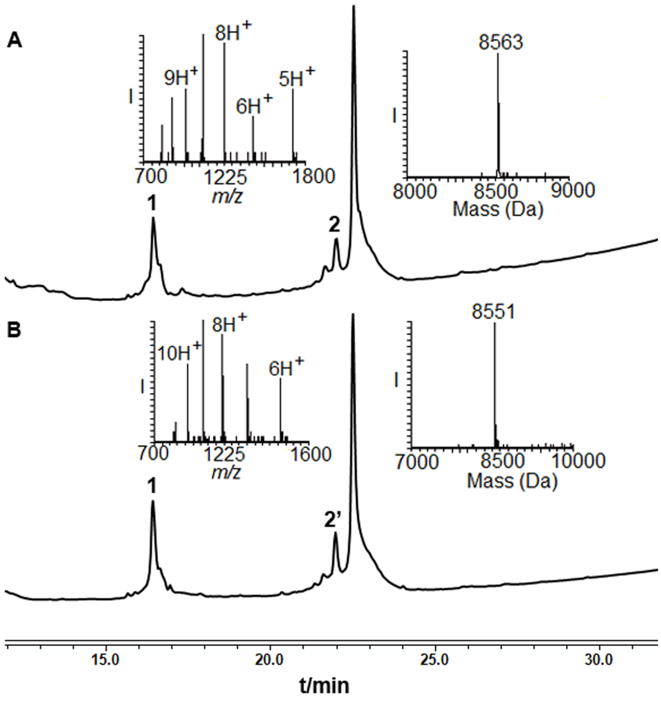

Supplementary Figure 17. HPLC and mass analyses of one-pot NCL and Thz opening. (A) NCL between CSP-1-1 and CSP-1-2: the main peak corresponds to the ligation product, with the observed mass 8563.0 Da ± 0.4 Da (calcd 8563.5 Da, average isotopes). (B) Thz opening using $[Pd(allyl)Cl]_2$/GSH the main peak corresponds to the Thz opening product, with the observed mass 8551.0 Da ± 0.4 Da (calcd 8551.5 Da, average isotopes). 1: CSP-1-1, 2: CSP-1-2, 2': hydrolyzed CSP-1-2.

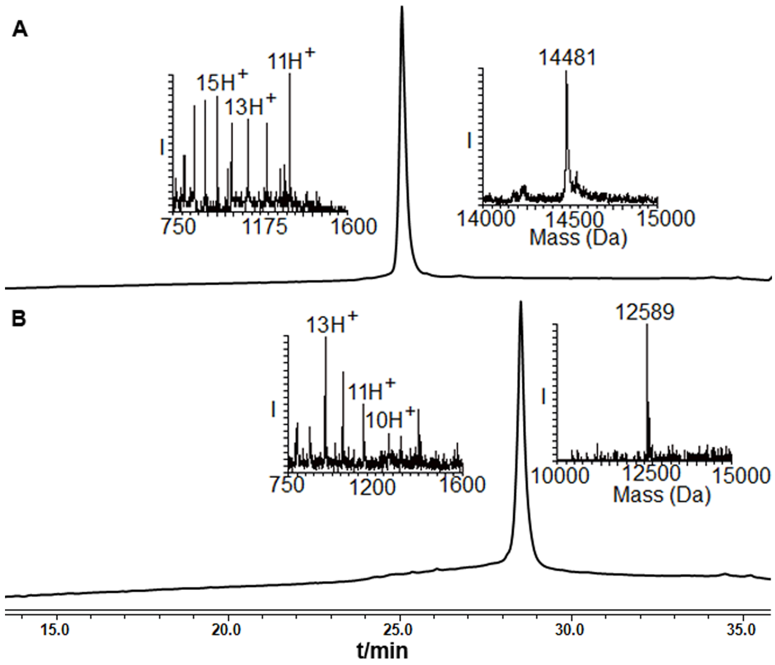

Supplementary Figure 18. NCL between CSP-1-3 and CSP-1-4, followed by tags removal. A) HPLC and mass analyses of the purified product of NCL between CSP-1-3 and CSP-1-4, with the observed mass 14481.0 Da ± 0.3 Da (calcd 14480.5 Da, average isotopes). B) HPLC and mass analyses of three tags removal via $PdCL_2$: the peak corresponds to the native Csp-1 with the observed mass 12589.0 Da ± 0.2 Da (calcd 12590.5 Da, average isotopes).

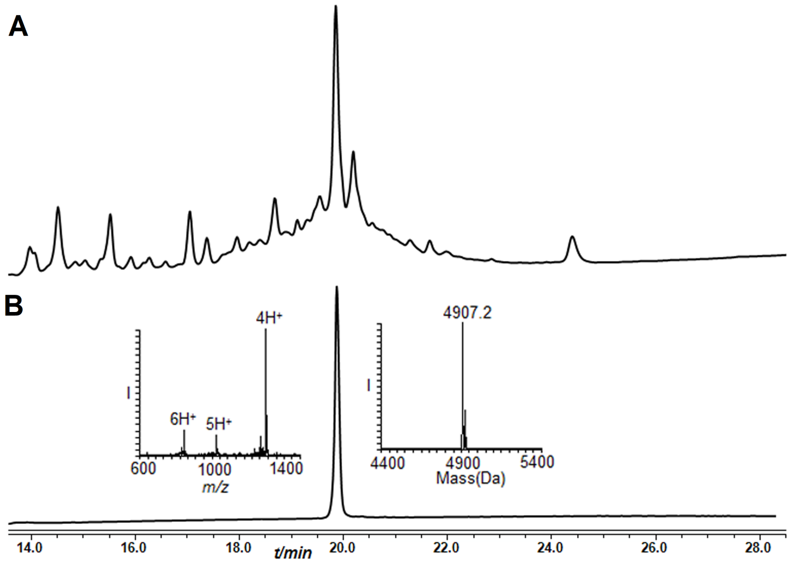

Supplementary Figure 19. HPLC and mass analyses of fragment H2A-1. (A) crude and (B) purified H2A-1, with the observed mass 4907.2 Da ± 0.2 Da (calcd 4906.7 Da, average isotopes).

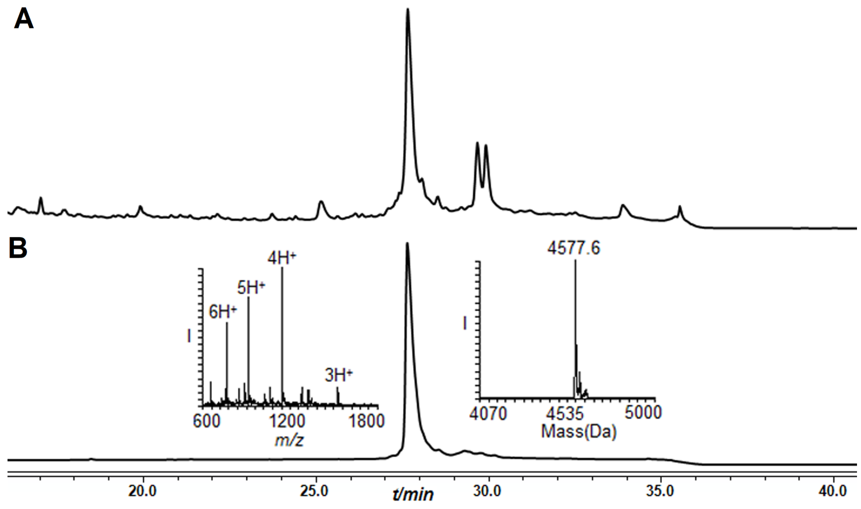

Supplementary Figure 20. HPLC and mass analyses fragment H2A-2. (A) crude and (B) purified H2A-2, with the observed mass 4577.6 Da ± 0.4 Da (calcd 4577.1 Da, average isotopes).

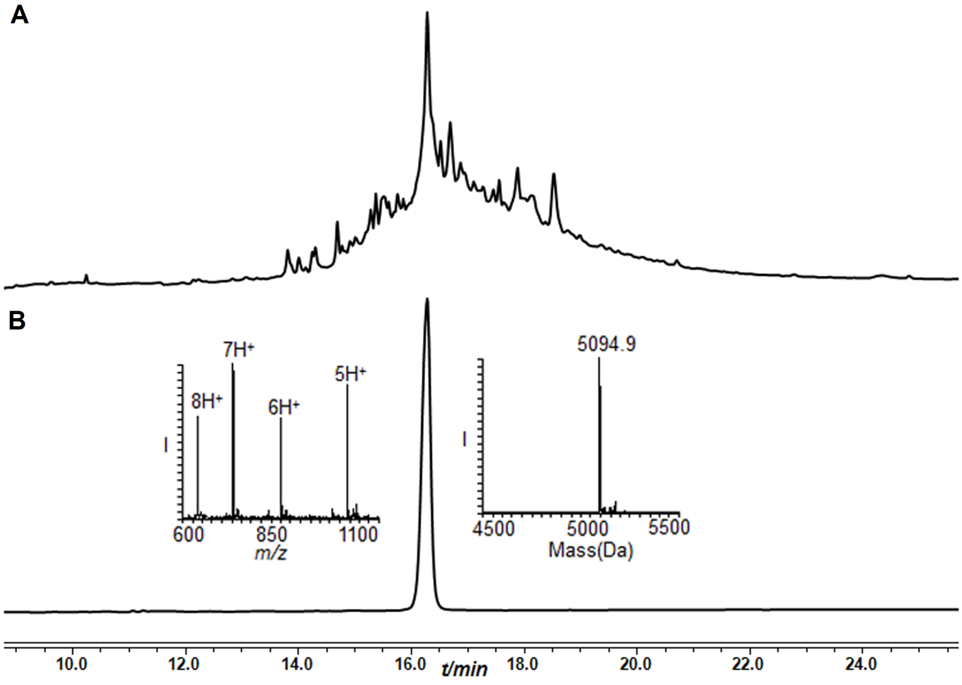

Supplementary Figure 21. HPLC and mass analyses of fragment H2A-3. (A) crude and (B) purified H2A-3, with the observed mass 5094.9 Da ± 0.4 Da (calcd 5094.6 Da, average isotopes).

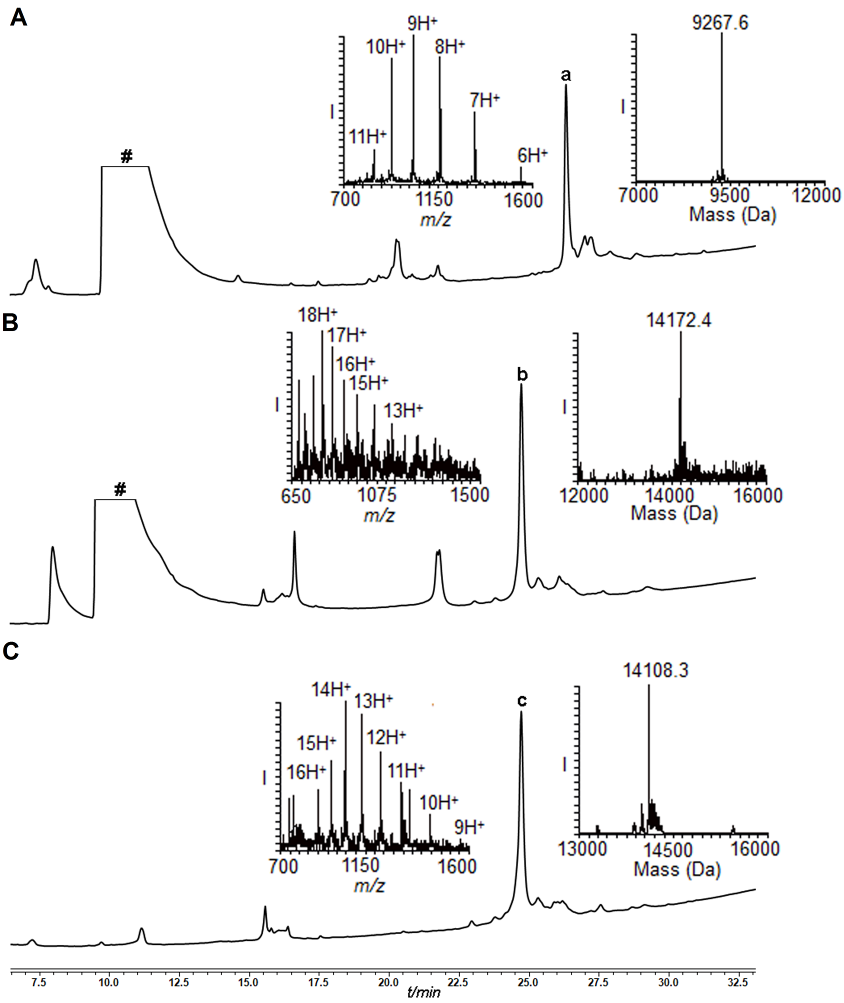

Supplementary Figure 22. One-pot chemical synthesis of H2A-5. (A) HPLC and mass analyses of NCL between H2A-1 and H2A-2: peak a corresponds to the ligation product and Thz removal with the observed mass 9267.6 Da ± 0.2 Da (calcd 9267.8 Da, average isotopes). (B) HPLC and mass analysis of the ligation product between H2A-3 and H2A-4, peak b corresponds to the ligation product with the observed mass 14172.4 Da ± 0.4 Da (calcd 14172.2 Da, average isotopes). (C) HPLC and mass analyses for the desulfurization reaction, peak c corresponds to the desulfurized product H2A-5 with the observed mass 14108.3 Da ± 0.2 Da (calcd 14108.2 Da, average isotopes). # is 4-mercaptophenylacetic acid (MPAA).

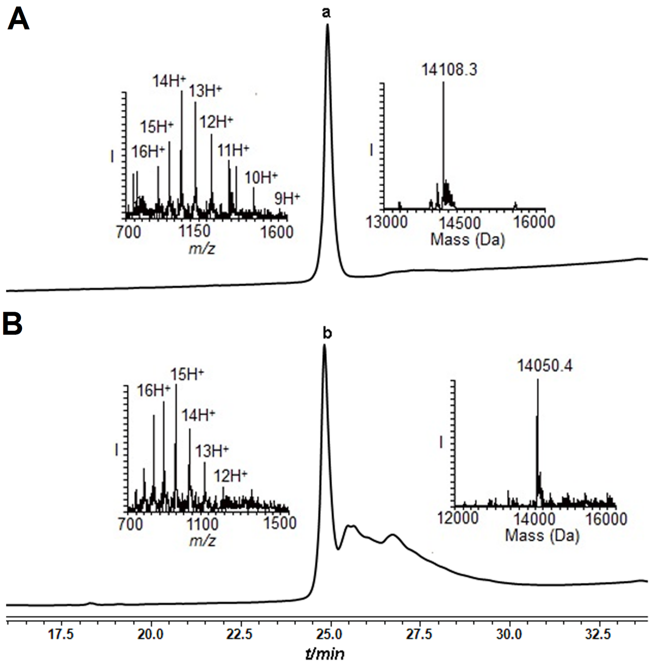

Supplementary Figure 23. HPLC and mass analyses of the *t*-Butyl removal from H2A-5 using Pd(II). (A) at time zero: peak a corresponds to the starting material (H2A-5) with the observed mass 14108.3 Da ± 0.1 Da (calcd 14108.2 Da, average isotopes). (B) After 2 h: peak b corresponds to the *t*-Butyl unmasked product with the observed mass 14050.4 Da ± 0.2 Da (calcd 14051.2 Da, average isotopes).

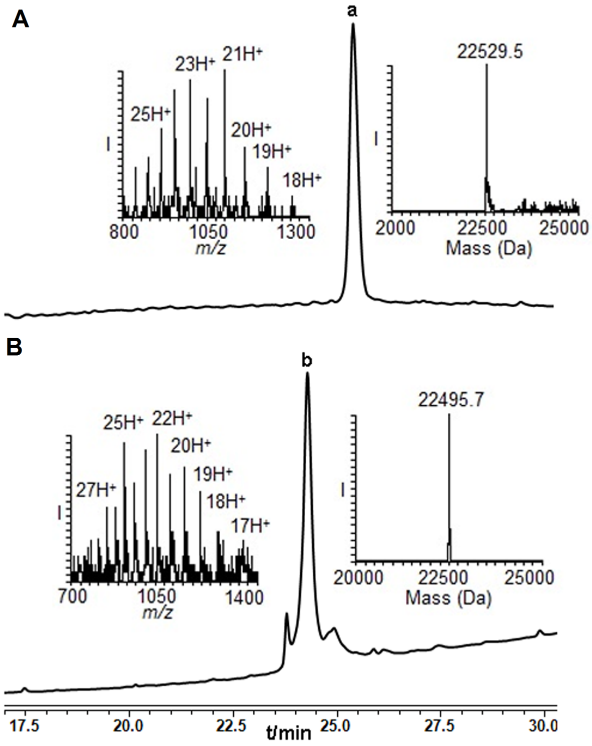

Supplementary Figure 24. HPLC and mass analyses of H2AK119$_{DHA}$Ub. (A) at time zero: peak a corresponds to the starting material with the observed mass 22529.5 Da ± 0.4 Da (calcd 22532 Da, average isotopes). (B) After 3 h: peak b corresponds to the H2AK119$_{DHA}$Ub product with the observed mass 22495.7 Da ± 0.2 Da (calcd 22498 Da, average isotopes).

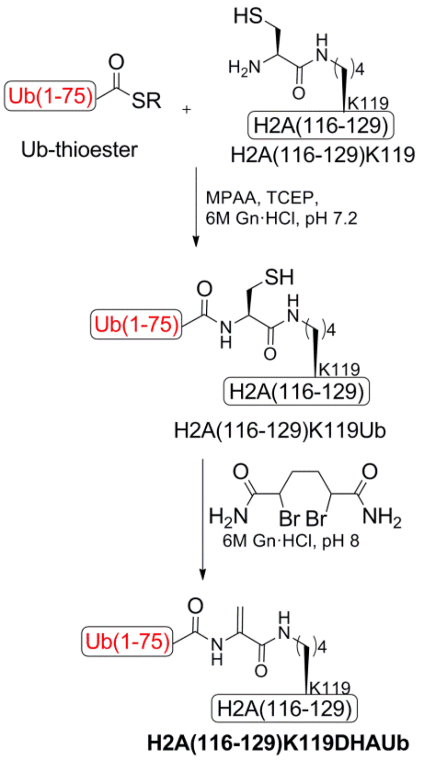

Supplementary Figure 25. Schematic representation of the Synthesis of H2A(116-129)K119$_{DHA}$Ub. The two peptide fragments were ligated employing NCL followed by Cys elimination to provide (H2A(116-129)K119$_{DHA}$Ub).

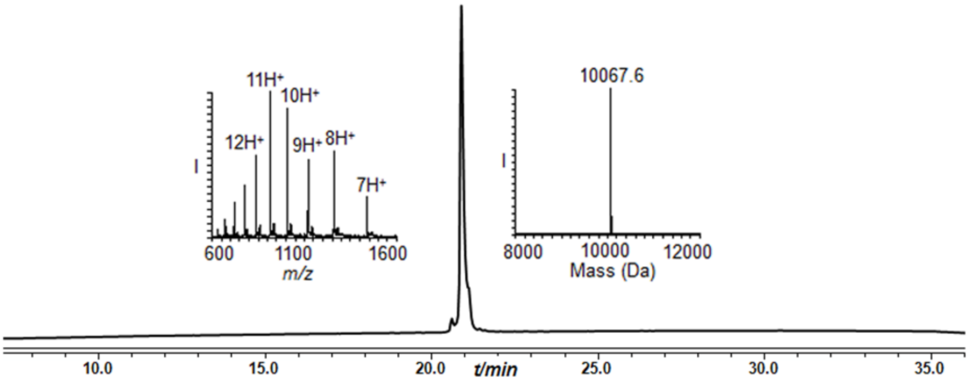

Supplementary Figure 26. HPLC and mass analyses of purified H2A(116-129)K119$_{DHA}$Ub. Observed mass 10067.6 Da ± 0.3 Da (calcd 10067.5 Da, average isotopes).

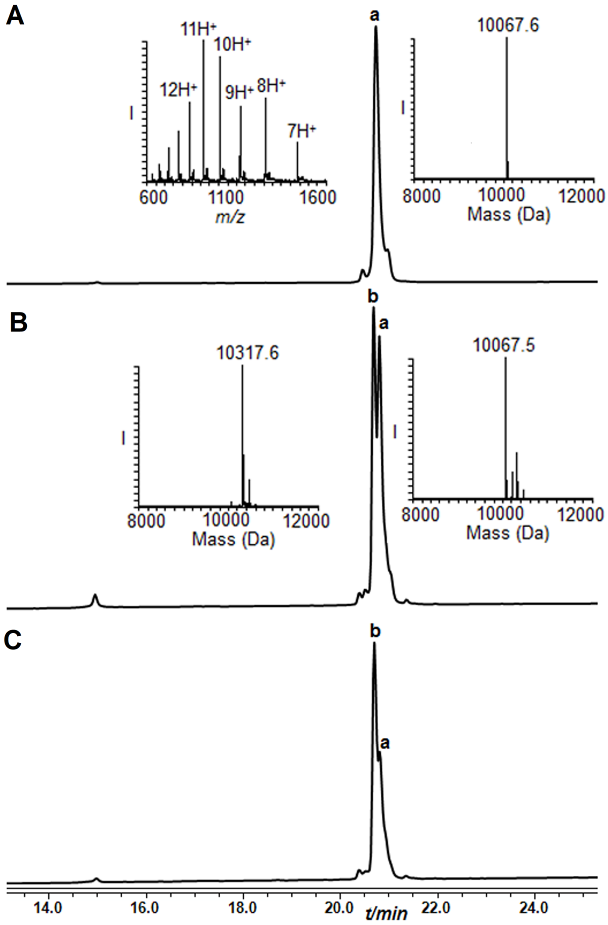

Supplementary Figure 27. HPLC and mass analyses of H2A(116-129)K119$_{DHA}$Ub in 10 mM Tris, 2 M NaCl, 1 mM EDTA, 1 mM TCEP buffer, pH~7.2 at 4°C. (A) at time zero: peak a corresponds to the starting material with the observed mass 10067.6 Da ± 0.3 Da (calcd 10067.5 Da, average isotopes). (B) After 24 h incubation: peak a corresponds to the starting material with the observed mass 10067.6 Da ± 0.3 Da (calcd 10067.5 Da, average isotopes) and peak b corresponds to the addition of TCEP molecule with the observed mass 10317.6 Da ± 0.2 Da (calcd 10317.5 Da, average isotopes). (C) After 48 h incubation.

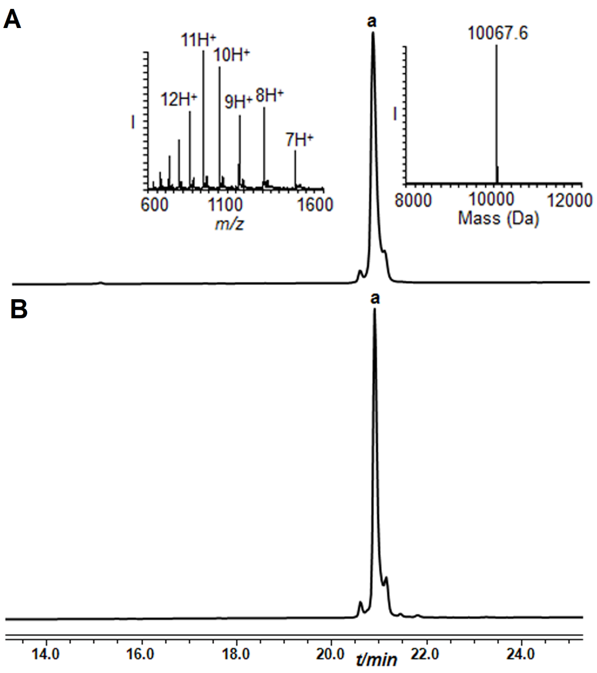

Supplementary Figure 28. HPLC and mass analysis in 10 mM Tris, 2 M NaCl, 1 mM EDTA buffer, pH~7.2 at 4°C. (A) at time zero: peak a corresponds to the starting material with the observed mass 10067.6 Da ± 0.3 Da (calcd 10067.5 Da, average isotopes). (B) After >20 days: peak a corresponds to the starting material with the observed mass 10067.6 Da ± 0.3 Da (calcd 10067.5 Da, average isotopes).

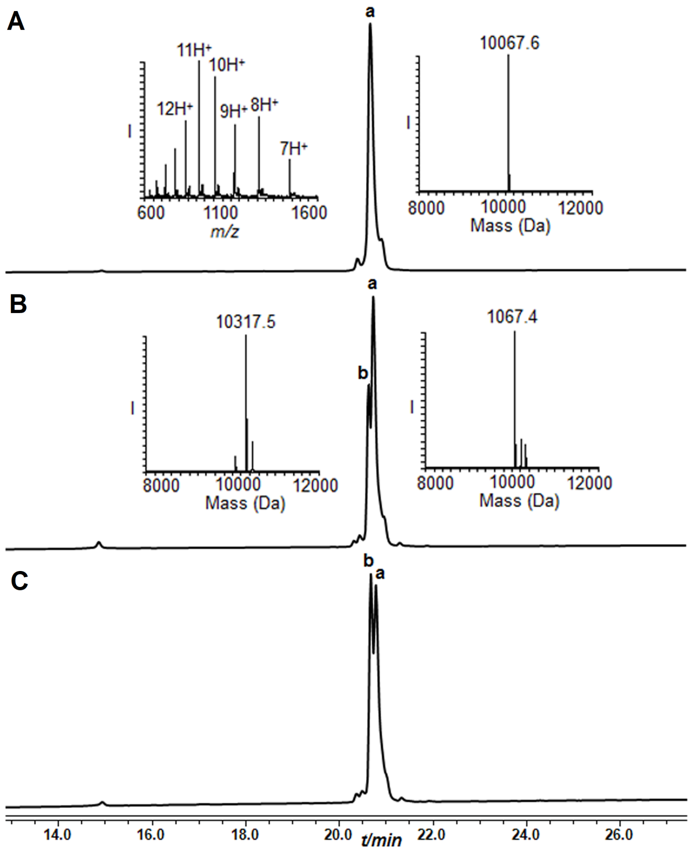

Supplementary Figure 29. HPLC and mass analysis of H2A(116-129)K119$_{DHA}$Ub in 10 mM HEPES, 2 M NaCl, 1 mM EDTA, 1 mM TCEP buffer, pH~7.2 at 4°C. (A) at time zero: peak a corresponds to the starting material with the observed mass 10067.6 Da ± 0.3 Da (calcd 10067.5 Da,

average isotopes). (B) After 24 h incubation: peak a corresponds to the starting material with the observed mass 10067.6 Da ± 0.3 Da (calcd 10067.5 Da, average isotopes) and peak b corresponds to the addition of TCEP with the observed mass 10317.6 Da ± 0.2 Da (calcd 10317.5 Da, average isotopes). (C) After 48 h incubation.

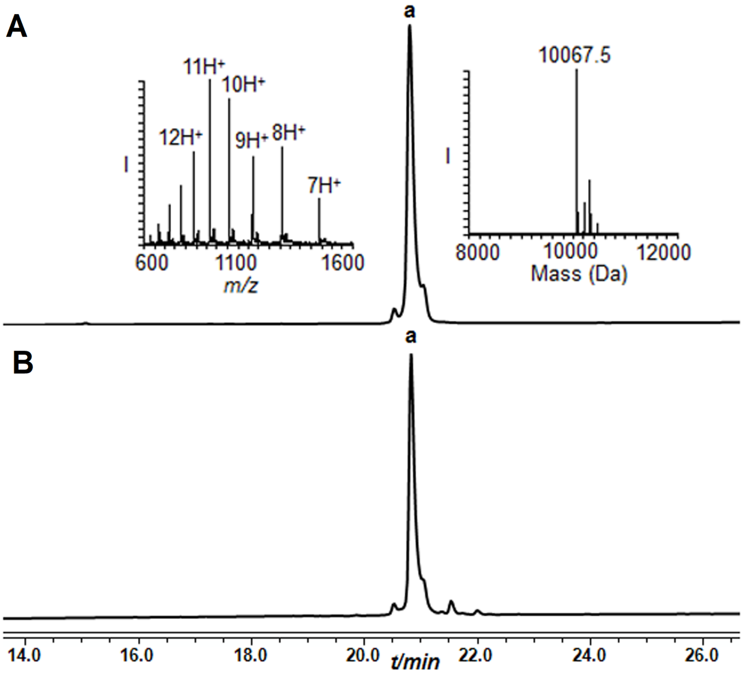

Supplementary Figure 30. HPLC and mass analysis in 10 mM HEPES, 2 M NaCl, 1 mM EDTA buffer, pH~7.2 at 4°C. (A) at time zero: peak a corresponds to the starting material with the observed mass 10067.6 Da ± 0.3 Da (calcd 10067.5 Da, average isotopes). (B) After >20 days: peak a corresponds to the starting material with the observed mass 10067.6 Da ± 0.3 Da (calcd 10067.5 Da, average isotopes).

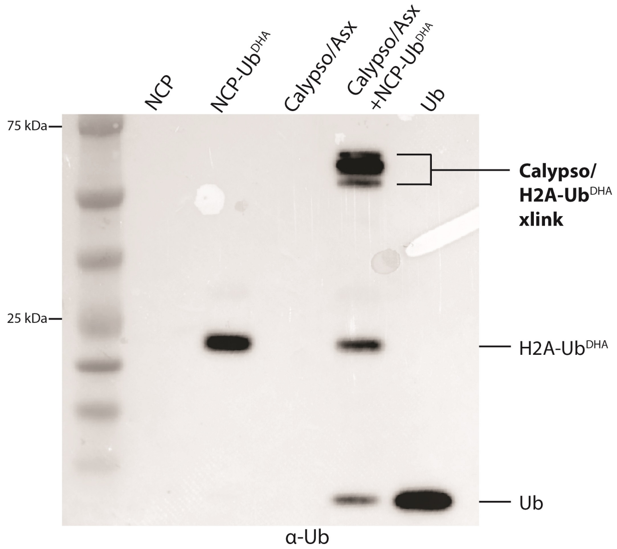

Supplementary Figure 31. Western blot analysis using ubiquitin antibody of the labeling reaction between NCP(H2A-Ub$_{DHA}$) and Calypso/ASX DUB. A new high-molecular-weight species, which contains ubiquitin formed upon reaction of Calypso/ASX with NCP(H2A-Ub$_{DHA}$).

Supplementary Tables

Supplementary Table 1. Stability of Cys(*t*-butyl) in peptide 2 to Pd(II) complexes under various conditions.

Entry	catalyst	equiv	buffer	additive	tempreture	time (h)	yield
1.	$[Pd(allyl)Cl]_2$	20	6M Gn.HCl	GSH	37°C	5	0%
2.	$[Pd(allyl)Cl]_2$	10	6M Gn.HCl	MPAA	37°C	5	0%
3.	$[Pd(allyl)Cl]_2$	10	6M Gn.HCl	TCEP	37°C	5	0%
4.	$[Pd(allyl)Cl]_2$	10	6M Gn.HCl	MPAA/TCEP	37°C	5	0%
5.	$[Pd(allyl)Cl]_2$	10	6M Gn.HCl	/	65°C	5	0%
6.	$PdCl_2$	20	6M Gn.HCl	GSH	37°C	5	0%
7.	$PdCl_2$	10	6M Gn.HCl	MPAA/TCEP	37°C	5	0%
8.	$PdCl_2$	10	6M Gn.HCl	TIS	37°C	5	0%
9.	$PdCl_2$	10	6M Gn.HCl	/	37°C	5	0%
10.	$PdCl_2$	10	6 MGn.HCl	/	65°C	5	0%
11.	$PdCl_2$	10	0.5M Gn.HCl	/	37°C	5	0%
12.	/	/	5M Urea	/	37°C	2	0%
13.	/	/	50mM Tris	/	37°C	2	0%

Supplementary Methods

General methods

SPPS was carried out manually in syringes, equipped with teflon filters, purchased from Torviq or by using an automated peptide synthesizer (CS336X, CSBIO). If not differently described, all reactions were carried out at room temperature. Analytical grade *N,N*-dimethylformamide (DMF) was purchased from Bio-Lab Ltd. Commercial reagents were used without further purification. Resins were purchased from Creosalus, protected amino acids were purchased from GL Biochem and activating reagents [(2-(1H-benzotriazol-1-yl)-1,1,3,3-tetramethyluronium hexafluorophosphate (HBTU), hydroxybenzotriazole (HOBt), [(6-chlorobenzotriazol-1- yl)oxy-(dimethylamino)methylidene]-dimethylazanium hexafluorophosphate (HCTU), 1- [Bis(dimethylamino)methylene]-1H-1,2,3-triazolo[4,5-b]pyridinium 3-oxid hexafluorophosphate (HATU)] were purchased from Luxembourg Bio Technologies. Analytical HPLC was performed on a Thermo instrument (Dionex Ultimate 3000) using analytical columns Xbridge (waters, BEH300 C4, 3.5µm, 4.6 × 150 mm) and XSelect (waters, CSH C18, 3.5 µm, 4.6 × 150 mm) at a flow rate of 1.2 mL/min. Preparative HPLC was performed on a Waters instrument using XSelect C18 10µm 19 × 250 mm and semi-preparative HPLC was performed on a Thermo Scientific instrument (Spectra System SCM1000) using Jupiter C4 10 µm, 300 Å, 250 × 10 mm column, at a flow rate of 15 and 4 mL/min respectively. All synthetic products were purified by HPLC and characterized by

mass spectrometry using LCQ Fleet Ion Trap (Thermo Scientific). All calculated masses have been reported as an average isotope composition. Buffer A: 0.1% TFA in water; buffer B: 0.1% TFA in acetonitrile.

List of the protected amino acids used in peptides synthesis

Fmoc-Gly-OH, Fmoc-Ala-OH, Fmoc-Val-OH, Fmoc-Leu-OH, Fmoc-Ile-OH, Fmoc-Phe-OH, Fmoc-Pro-OH, Fmoc-His(Trt)-OH, Fmoc-Asn(Trt)-OH, Fmoc-Gln(Trt)-OH, Fmoc-Arg(Pbf)-OH, Fmoc-Lys(Boc)-OH, Fmoc-Tyr(tBu)-OH, Fmoc-Ser(tBu)-OH, Fmoc-Thr(tBu)-OH, Fmoc-Asp(OtBu)-OH, Fmoc-Glu(OtBu)-OH, Fmoc-Trp(Boc)-OH, Fmoc-Met-OH, Fmoc-Cys(Acm)-OH, Fmoc-Cys(*t*-Butyl)-OH, Boc-Cys(Trt)-OH, Boc-Thz-OH, Boc-Arg(Pbf)-OH, Fmoc-Leu-Thr(ψMe,MePro)-OH, Fmoc-Lys(Boc)-Thr(ψMe,MePro)-OH, Fmoc-Val-Thr(ψMe,MePro)-OH. Fmoc-Asp(OtBu)-(Dmb)Gly-OH, Fmoc-Ile-Thr(ψMe,MePro)-OH, Fmoc-Leu-Ser(ψMe,MePro)-OH.

H2A sequence

SGRGKQGGKTRAKAKTRSSRAGLQFPVGRVHRLLRKGNYAERVGAGAPVYLAAVL
EYLTAEILELAGNAARDNKKTRIIPRHLQLAVRNDEELNKLLGRVTIAQGGVLPNIQS
VLLPKKTESSKSAKSK

CSP-1 sequence

GEDPHAGHKMSHGAKYKALLDSSSHCVAVGEDCLRHCFEMLAMNDASMGACTKA
TYDLVAACGALAKLAGTNSAFTPAFAKVVADVCAACKKECDKFPSIAECKACGEAC
QACAEECHKVAA

Synthesis of peptide 1

The synthesis of Thz-LYRAGC(Acm)LYRAG was carried out using Fmoc-SPPS on Rink amide resin (0.43 mmol/g, 0.2 mmol scale). Peptide synthesis was performed on peptide synthesizer in presence of 4 equiv. of amino acid, HCTU and 8 equiv. of *N,N'*-diisopropylethylamine (DIEA). For the synthesis of the model peptides, the pre-swollen resin was treated with 20% piperidine in DMF containing 0.1 mmol HOBt (3-5-3 min) to remove the Fmoc-protecting group.

Deprotection and cleavage from the resin: The resin was washed with DMF, MeOH, DCM and dried. The peptide was cleaved using Trifluoroacetic acid (TFA):triisopropylsilane (TIS):water (95:2.5:2.5) cocktail for 2 h. The cleavage mixture was filtered and the combined filtrate was added dropwise to a 10-fold volume of cold ether and centrifuged. The precipitated crude peptide was dissolved in 50% acetonitrile/water and lyophilized. The

HPLC analysis was carried out on a C18 analytical column using a gradient of 0-60% B over 30 min. For preparative HPLC, C18 column in gradient of 0-40% B was used to provide peptide 1 in ~50% yield.

Thz removal using $PdCl_2$ GSH(1:1) in the presence of Acm

0.5 mg of peptide 1 (0.35×10^{-3} mmol, 2mM) was dissolved at 175 µL, H_2O, and treated with 10 equiv. of $PdCl_2$ and GSH (1:1) and incubated at 37°C for overnight.

Synthesis of peptide 2

The synthesis of AC(*t*-butyl)LYRAGLYRAG was carried out using Fmoc-SPPS as described in peptide 1. The peptide was cleaved from the resin as described above and lyophilized. The HPLC analysis was carried out on a C4 analytical column using a gradient of 0-60% B over 30 min. For preparative HPLC, C18 column in gradient of 0-40% B was used to provide peptide 2 in ~55% yield.

Unmasking Cys(*t*-butyl) in peptide 2 using palladium under various conditions

0.5 mg of peptide 2 (0.36×10^{-3} mmol, 2mM) was dissolved at 180 µL, under various buffers, and treated with 10 equiv. of $PdCl_2$ or $[Pd(allyl)Cl]_2$ with deferent additives and incubated at 37°C for different time course.

For a complete *t*-butyl deprotection 0.5 mg of peptide 2 (0.36×10^{-3} mmol, 2mM) was dissolved in 180 µL, 50mM Tris or 5 M Urea buffers and treated with 10 equiv. of $PdCl_2$ at 37°C for 1-2 h.

Synthesis of peptide Thz-LYRAC(Acm)LYRAC(*t*-butyl)LYRAG

The synthesis of the peptide was carried out using Fmoc-SPPS on Rink amide resin (0.43 mmol/g, 0.1 mmol scale). was carried out using Fmoc-SPPS as described in peptide 1. The HPLC analysis was carried out on a C4 analytical column using a gradient of 0-60% B over 30 min. For preparative HPLC, C18 column in gradient of 0-40% B was used to provide Thz-LYRAC(Acm)LYRAC(t-Butyl)LYRAG peptide in ~45% yield.

Orthogonality between Thz, Acm and *t*-butyl in a model peptide

1 mg of peptide; Thz-LYRAC(Acm)LYRAC(*t*-butyl)LYRAG (0.5×10^{-3} mmol, 2mM) was dissolved in 250 μL, 6 M Gn.HCl, pH 7.3, and treated with 10 equiv. of $[Pd(allyl)Cl]_2$ and GSH (1:1) and incubated at 37°C for 45 min to observe complete Thz removal. Subsequently, the addition of extra 10 equiv. of $[Pd(allyl)Cl]_2$ to the reaction mixture, enabled complete Acm removal within 5 h.

Synthesis of peptide 3

The synthesis of Thz-LYRAGC(Acm)LYRCA was carried out using Fmoc-SPPS as described in peptide 1. The HPLC analysis was carried out on a C4 analytical column using a gradient of 0-60% B over 30 min. For preparative HPLC, C18 column in gradient of 0-40% B was used to provide peptide 3 in ~50% yield.

Palladium scavenger resin

Preparation: PEGA resin (0.2 mmol/g, 0.1 mmol scale) was swelled in DMF, then Fmoc-Ala-OH and Boc-Cys(Trt)-OH, were coupled to the resin using 4 eq HATU, 8 equiv. DIEA, for 1 h for each coupling. Finally, the resin was treated with cleavage mixture for 1 h to afford the PEGA resin with free cysteine. Then the resin was washed carefully with DMF, water and 6 M Gn.HCl, 200 mM phosphate buffer, pH ~7.3 and kept in the Gn.HCl buffer at room temperature.

For scavenging excess of Pd(II), 4 equiv. of resin to the Pd(II) was incubated with the reaction mixture in centrifugal filters (0.22μm) and shacked for 1 h at 37 ºC followed by separation of the resin from the reaction mixture. Similar procedure was performed in order to cap excess of the alkylating reagents: (*N*-(bromoacetyl)alanine-methyl ester, *N*-(bromoacetyl)glycine-ethyl ester and iodoacetamide) from the reaction mixture.

Inverse Sequential modification of peptide 3

2 mg of peptide 3 (1.35×10^{-3} mmol, 2.7mM) was dissolved at 500 µL, 20 % CH_3CN/H_2O, and treated with 1 equiv. of *N*-(bromoacetyl)glycine-ethyl ester, which was pre-dissolved as a stock solution in MeOH with 100 equiv. of NaI and added to the reaction mixture. The reaction was monitored by HPLC-MS, which showed complete reaction within 30 min. Then, the reaction mixture was treated with 10 equiv. of $PdCl_2$ and incubated at 37°C for 30 min, followed by DTT treatment to provide complete Acm deprotection to provide mono-alkylated peptide 3.

1 mg of mono-alkylated peptide 3 (0.6×10^{-3} mmol, 2mM) was dissolved at 320 µL, 6 M Gn.HCl, pH 8, and treated with 1 equiv. of *N*-(bromoacetyl)alanine-methyl ester, which was pre-dissolved in MeOH, and the reaction mixture was incubated at 37°C for 30 min. For the following Thz removal, 10 equiv. of $[Pd(allyl)Cl]_2$ and GSH (1:1) were added to the reaction and incubated at 37°C for 45 min to observe complete Thz opening. The solution mixture was treated with 40 equiv. Cys-PEGA resin for 1 h at 37°C and 1 equiv. of DTT. Finally, 15 equiv. of iodoacetamide at 37°C were added to give the tri-alkylated peptide 3.

Synthesis of CSP-1-1 bearing a solubilizing tag

The synthesis was carried out using Fmoc-SPPS on Rink amide resin (0.43 mmol/g, 0.1 mmol scale). The pre-swollen resin was treated with 20% piperidine in DMF containing 0.1 mmol HOBt (3-5-3 min) in order to remove the Fmoc-protecting. Peptide synthesis was performed on an automated peptide synthesizer in presence of 4 equiv. of amino acid, HCTU and 8 equiv. DIEA. The Phacm linker was coupled manually in position 116, using 2.5 equiv. of the linker, HATU and 5 equiv. DIEA for 1.5 h. Alloc deprotection from the linker was carried out by using 0.2 equiv. tetrakis(triphenylphosphine)palladium(0) ($Pd(PPh_3)_4$) and 20 equiv. phenylsilane in dry DCM for 1 h. Fmoc-Arg(Pbf) was coupled to the linker using HATU as a coupling agent. This step was repeated to couple the second Arg. The third Arg of the tag was coupled as Boc-Arg(Pbf)-OH. For side chains deprotection and cleavage from the resin, the solid support was washed with DMF, MeOH, DCM and dried. Then the resin was treated

with the cleavage mixture containing TFA:TIS:water (95:2.5:2.5) for 2 h. The cleavage mixture was filtered and the combined filtrate was added dropwise to a 10-fold volume of cold ether and centrifuged. The precipitated crude peptide was dissolved in 50% acetonitrile/water and lyophilized. HPLC analysis was carried out on a C4 analytical column using a gradient of 0-60% B over 30 min. For preparative HPLC, C18 column in gradient of 20-60%B was used to purify CSP-1-1 in ~35% yield.

Synthesis of CSP-1-2 bearing solubilizing tag

This peptide was prepared using Fmoc-amino-4-(methylamino)benzoic acid linker (Fmoc-N-MeDbz-OH). The Fmoc-N-MeDbz-OH was coupled manually on pre-swollen Rink amide resin (0.1 mmol) using 4 equiv. amino acid, HATU and 8 equiv. DIEA for 1.5 h. The remaining amino acids were coupled on an automated peptide synthesizer using 4 equiv. amino acid, HCTU and 8 equiv. of DIEA. The Phacm linker was coupled manually in position 63 using 2.5 equiv. of linker, HATU and 5 equiv. DIEA for 1.5 h. Alloc deprotection from the linker and 3 Arg coupling was carried out as described above. N-*MeDbz cyclization:* The resin was washed with DCM and a solution of *p*-nitrophenyl chloroformate (120 mg, for 0.1 mmol scale) in 4 mL DCM was added and shaken for 30 min at room temperature and washed with DCM (3 × 5 mL). This step was repeated twice. Following this, the resin was treated with a solution of DIEA (0.45mL) in DMF (4 mL) and was shaken for 30 min for a complete cyclization and washed with DMF (×2). Analytical HPLC analysis was performed to ensure complete reactions. The peptides were cleaved from the resin as described above and lyophilized.

MMP switching: The crude cyclized peptide was dissolved in 6 M Gn.HCl, 200 mM phosphate buffer at pH 7.3 and treated with 200 equiv. of MMP at 37 ^{o}C for 2 h. The progress of the reaction was monitored by analytical HPLC (C4 column), using a gradient of 0-60% B over 30 min. For preparative HPLC, C18 column in gradient of 20-60%B was used to purify CSP-1-2 in ~40% yield.

Synthesis of CSP-1-3 bearing solubilizing tag

This peptide was prepared by using the same procedure described for CSP-1-2. The HPLC analysis was carried out on a C4 analytical column using a gradient of 0-60% B over 30 min. For preparative HPLC, C18 column in gradient of 20-60%B was used to purify CSP-1-3 in ~25% yield.

Circular dichroism spectroscopy of purifies full-length CSP-1:

CSP-1 was dissolved in 6 M urea solution (5% of the total volume) and then further diluted with 20 mM Tris buffer solution and pH was adjusted to 7.3 . The exact concentration of the protein solution was determined by Pierce® BCA Protein Assay Kit, Thermo scientific. With this solution circular dichroism spectrum was recorded in a Chirascan (Applied Photophysics) instrument.

Synthesis of H2A-1 [Cys-H2A(87-129)]

The synthesis of H2A-1 [Cys-H2A(87-129)] was carried out on a Rink amide resin (0.43 mmol/g, 0.1 mmol scale). The pre-swelled resin was treated with 20% piperidine in DMF containing 0.1 mmol HOBt (3-5-3 min) for Fmoc removal. The amino acids were coupled in an automated peptide synthesizer using 4 equiv. of amino acid, 8 equiv. of DIEA and 4 equiv. of HCTU to the initial loading of the resin. N^{α}-Fmoc-N^{ε}-Alloc-Lys-OH was coupled at position Lys119 of the H2A sequence. Ala 87 was mutated to free Cys to enable native chemical ligation. Pseudoproline dipeptides Fmoc-Lys(Boc)-Thr(ψMe,MePro)-OH and Fmoc-Val-Thr(ψMe,MePro)-OH were coupled manually at positions Lys120-Thr121 and Val101-Thr102, respectively, using 2.5 equiv. of dipeptides, 2.5 equiv. of HATU and 5 equiv. of DIEA for 1.5 h. The Alloc protecting group was removed using 0.2 equiv. of ($Pd[P(C_6H_5)_3]_4$) and 20 equiv. phenylsilane in dry DCM. Then Fmoc-Cys(*t*-Butyl)-OH was coupled manually. Analytical HPLC analysis was performed to ensure complete reactions. The peptide was deprotected and cleaved from the resin as described above. The HPLC analysis was carried out on a C18 analytical column and a gradient of 0-60% B over 30 min.

For preparative HPLC, C18 column with the same gradient was used to purify the H2A-1 in ~40% yield.

Synthesis of H2A-2 [Thz-H2A(48-85)-Nbz]

The synthesis of H2A-2 was carried out on Rink amide resin (0.43 mmol/g, 0.1 mmol scale). Unless specified otherwise, for each coupling cycle, amino acids and HCTU were used in 4-fold excess, while DIEA was used in 8-fold excess to the initial loading of the resin. The pre-swelled resin was treated with 20% piperidine in DMF containing 0.1 mmol HOBt (3-5-3 min) to remove the Fmoc protecting group. Fmoc-3,4-diaminobenzoic acid (Fmoc-Dbz) was coupled to the resin using HBTU/HOBt for 1 h, (2 cycles). Subsequently, the resin was washed with DMF (3 × 5 mL). Following Fmoc removal, the Leu86 was manually coupled using 6 equiv. of amino acid, 6 equiv. HCTU and 9 equiv. DIEA for 1 h (2 cycles). The remaining amino acids were coupled manually. Pseudoproline dipeptides Fmoc-Leu-Thr(ψMe,MePro)-OH and Fmoc-Lys(Boc)- Thr(ψMe,MePro)-OH were coupled at positions Leu59-Thr60 and Lys76-Thr77, respectively, by using 2.5 equiv. of dipeptides, 2.5 equiv. of HATU and 5 equiv. of DIEA for 1.5 h. The last three amino acids i.e. Boc-Thz-OH, Fmoc-Pro-OH and Fmoc-Val-OH were coupled twice using HATU/DIEA for 1 h. Analytical HPLC analysis was performed to ensure complete reactions. Then, the resin was treated with a solution of p-nitrophenyl chloroformate (100 mg, 5 equiv.) in 4 mL of dry DMF and shaken for 30 min at room temperature and washed with DMF (3 × 5 mL). This step was repeated twice. Following this, the resin was treated with a solution of 0.5 M DIEA in DMF (4 mL) and was shaken for 30 min for complete cyclization and washed with DMF. Subsequently, the peptide was cleaved similarly as described above. The HPLC analysis was carried out on a C18 analytical column and a gradient of 0-60% B over 30 min. For preparative HPLC, C18 with the same gradient was used to purify the H2A-2 in ~40% yield.

The synthesis of H2A-3 [H2A(1-46)-N-MeNbz]

The synthesis was carried out using Rink amide resin (0.43 mmol/g, 0.1 mmol scale). The preswollen resin was treated with 20% piperidine in DMF containing 0.1 mmol HOBt (3-5-3 min) to remove the Fmoc-protecting group. Fmoc-*N*-MeDbz-OH was activated with HATU/DIEA and was coupled to the resin for 1 h. Subsequently, the resin was washed with DMF (3 × 5 mL). Following the Fmoc removal, Gly46 was manually coupled using 4 equiv. amino acid, 4 equiv. HATU and 8 equiv DIEA for 1h (2 cycles). The remaining amino acids were coupled on an automated peptide synthesizer in presence of 4 equiv. of amino acid, 4 equiv. of HCTU and 8 equiv. of DIEA to the initial loading of the resin. Pseudoproline dipeptide FmocLys(Boc)-Thr(ψMe,MePro)-OH was coupled manually at positions Lys10-Thr11 and Lys16-Thr17, by using 2.5 equiv. of dipeptides, 2.5 equiv. of HATU and 5 equiv. of DIEA for 1.5 h. N-MeDbz cyclization was performed as described above. The peptides were cleaved from the resin as described above and lyophilized. For preparative HPLC, C18 with the same gradient was used to purify the H2A-3 in ~40% yield.

H2A(116-129)K119$_{DHA}$Ub synthesis

H2A(116-129)K119, (3 mg, 1.8 × 10^{-3} mmol) and Ub(1-75)-thioester, (17.5 mg, 2 × 10^{-3} mmol), were dissolved in 6 M Gn·HCl, 200 mM Na_2HPO_4 buffer, pH ~7.2 (450 μL, 4 mM), containing 40 equiv. of MPAA and 20 equiv. of TCEP. The reaction was incubated at 37 ^{o}C for 30 min. The progress of the reaction was monitored by analytical HPLC using C4 analytical column and a gradient of 0-60% buffer B over 30 min. After completion of the reaction, the product (H2A(116-129)K119Ub) was isolated via semi-preparative HPLC, C4 column and a gradient of 20-60%B to isolate the product in ~60% yield. 100 equiv. of bisamide1,4-dibromobutane dissolved in DMF were added H2A(116-129)K119Ub, (4 mg, 3.9 × 10^{-4} mmol) dissolved in (200 μL, 2 mM) in 6 M Gn·HCl, 200 mM Na_2HPO_4 buffer, pH ~8 and the reaction mixture was incubated 1 h at room temperature, followed by 3 h at 37 ºC. The progress of the reaction was monitored by analytical HPLC using C4 analytical column and a gradient of 0-60% buffer B over 30 min. After completion of the reaction, the product

(H2A(116-129)K119$_{DHA}$Ub) was isolated via semi-preparative HPLC, C4 column in gradient of 20-60%B was used to isolate the product in ~60% yield.

H2A(116-129)K119$_{DHA}$Ub stability

H2A(116-129)K119$_{DHA}$Ub probe (2 mg, 9.8×10^{-3} mmol) was dissolved in a minimum amount of 6 M Gn·HCl, 200 mM Na_2HPO_4 buffer, pH ~7.2 and incubated separately to various conditions including: different buffers (e.g. Tris and HEPES), additive (e.g. TCEP) and temperatures (4 ^{o}C, RT and 37 ^{o}C) for different times course to study the probe stability under these conditions.
